# Overexpression of *AcWRKY31* Increases Sensitivity to Salt and Drought and Improves Tolerance to Mealybugs in Pineapple

**DOI:** 10.3390/plants13131850

**Published:** 2024-07-05

**Authors:** Myat Hnin Wai, Tiantian Luo, S. V. G. N. Priyadarshani, Qiao Zhou, Mohammad Aqa Mohammadi, Han Cheng, Mohammad Aslam, Chang Liu, Gaifeng Chai, Dongping Huang, Yanhui Liu, Hanyang Cai, Xiaomei Wang, Yuan Qin, Lulu Wang

**Affiliations:** 1 College of Agriculture, Fujian Provincial Key Laboratory of Haixia Applied Plant Systems Biology, Pingtan Science and Technology Research Institute, Fujian Agriculture and Forestry University, Fuzhou 350002, China; myathninwai.edu@gmail.com (M.H.W.); luotiantian1022@163.com (T.L.); zhouqiao0606@163.com (Q.Z.); mohammadaqam85@gmail.com (M.A.M.); c.hanngu922@gmail.com (H.C.); aslampmb1@gmail.com (M.A.); changliu08@163.com (C.L.); 15117155242@163.com (G.C.); z35068120000419@163.com (D.H.); yanhuiliu520@gmail.com (Y.L.); caihanyang123@163.com (H.C.); wangxiaomei159@163.com (X.W.); 2 College of Life Science, Fujian Provincial Key Laboratory of Haixia Applied Plant Systems Biology, Pingtan Science and Technology Research Institute, Fujian Agriculture and Forestry University, Fuzhou 350002, China; 3 College of Horticulture, Fujian Provincial Key Laboratory of Haixia Applied Plant Systems Biology, Pingtan Science and Technology Research Institute, Fujian Agriculture and Forestry University, Fuzhou 350002, China; 4 Department of Botany, Mandalay University of Distance Education, Ministry of Education, Mandalay 05024, Myanmar; 5 Department of Applied Sciences, Faculty of Humanities and Sciences, Sri Lanka Institute of Information Technology, New Kandy Road, Malabe 10115, Sri Lanka; nirosha.p@sliit.lk; 6 Horticulture Research Institute, Guangxi Academy of Agricultural Sciences, Nanning Investigation Station of South Subtropical Fruit Trees, Ministry of Agriculture, Nanning 530007, China

**Keywords:** pineapple, WRKY transcription factor, *AcWRKY31*, biotic stress, abiotic stress

## Abstract

Pineapple is a globally significant tropical fruit, but its cultivation faces numerous challenges due to abiotic and biotic stresses, affecting its quality and quantity. WRKY transcription factors are known regulators of stress responses, however, their specific functions in pineapple are not fully understood. This study investigates the role of *AcWRKY31* by overexpressing it in pineapple and *Arabidopsis*. Transgenic pineapple lines were obtained using *Agrobacterium*-mediated transformation methods and abiotic and biotic stress treatments. Transgenic *AcWRKY31-OE* pineapple plants showed an increased sensitivity to salt and drought stress and an increased resistance to biotic stress from pineapple mealybugs compared to that of WT plants. Similar experiments in *AcWRKY31-OE*, *AtWRKY53-OE*, and the *Arabidopsis* Atwrky53 mutant were performed and consistently confirmed these findings. A comparative transcriptomic analysis revealed 5357 upregulated genes in *AcWRKY31-OE* pineapple, with 30 genes related to disease and pathogen response. Notably, 18 of these genes contained a W-box sequence in their promoter region. A KEGG analysis of RNA-Seq data showed that upregulated DEG genes are mostly involved in translation, protein kinases, peptidases and inhibitors, membrane trafficking, folding, sorting, and degradation, while the downregulated genes are involved in metabolism, protein families, signaling, and cellular processes. RT-qPCR assays of selected genes confirmed the transcriptomic results. In summary, the *AcWRKY31* gene is promising for the improvement of stress responses in pineapple, and it could be a valuable tool for plant breeders to develop stress-tolerant crops in the future.

## 1. Introduction

Plant breeders are currently facing a huge challenge developing new, high-yielding, and stress-tolerant crop varieties that can cope with environmental cues. Plants encounter various stresses throughout their life cycle, such as salinity, drought, temperature fluctuations, and pathogen infections [1]. To adapt to challenging environments, plants attempt to regulate their physiological and biochemical mechanisms in a timely manner [2,3]. At the same time, plant breeders are working on the development of stress-tolerant varieties to meet global demand.

The detrimental effects of abiotic stress on plants primarily arise from unfavorable alterations in the natural environment due to changes in temperature, salinity, etc. These changes promote corresponding physiological and biochemical responses in plants, ultimately causing significant harm to their inherent traits and phenotypic characteristics, leading to crop losses [4]. High levels of plant osmotic pressure and high levels of salinity can impair plant growth and development [5]. Drought is another significant factor that exerts detrimental effects on plant processes [6]. Additionally, drought stress has been associated with increased plant root biomass [7]. When the water content decreases in plant environments, the water potential in leaves also declines, which impairs photosynthesis and metabolism, and, in severe cases, leads to plant mortality [8]. Biotic stress, primarily caused by harmful pathogens, can penetrate plant cuticles, invade stomata, or exploit natural wounds to disrupt normal organ functions, leading to reduced photosynthetic rates, water imbalances, and nutrient transport obstacles [9,10].

*Ananas comosus* (L.) Merr. (pineapple) is an economically important crop that is distinctly exposed to disease conditions caused by bacteria, fungi, viruses, and nematodes, as well as abiotic stresses, leading to yield reductions [11]. *Dysmicoccus brevipes* (pineapple mealybug) is one of the most serious pests, and it weakens plants by sap-sucking and acts as a vector for the pineapple mealybug wilt-associated virus (PMWaV) [12]. Another destructive pathogen, *Sclerotinia sclerotiorum*, infects over 400 plant species, impacting crop yield and quality [13]. These pathogens pose a significant threat to various plants, including dicotyledons, such as sunflower, soybean, and rape, as well as monocotyledons, like onion and tulip [14]. Consequently, advanced biotechnologies play an essential role in the improvement of plant resistance to environmental stresses, identifying stress-resistant genes and facilitating the development of new plant varieties.

Among the transcription factor (TF) families involved in stress regulation, WRKY TFs are known for their diverse regulatory mechanisms. Typical WRKY proteins efficiently bind to W-box elements to modulate downstream gene transcription and can form protein complexes by interacting with other activated elements, enhancing their transcriptional binding ability. Structurally, WRKY TFs consist of two main domains: the DNA-binding N-terminal domain and the C-terminal zinc finger structure [15]. The WRKY DNA binding domain exhibits variations within the family according to the basic features of conservative heptapeptides, such as WRKYGMK, WRKYGKK, WRKYGQK, WSKYGQK, WKRYGQK, WVKYGQK, and WKKYGQK [16]. Zinc finger structures are primarily composed of C_2_H_2_ and C_2_HC motifs and are crucial constituents of WRKY TFs. WRKY TFs are classified into subfamilies I, II, and III by the number of their domains and their zinc finger-like structure [17]. The data from an evolutionary analysis showed that subfamily II can divide into sub-subfamilies, like IIa, IIb, IIc, IId, and IIe [18]. Furthermore, some WRKY families also contain the proline enrichment domain, glutamate enrichment domain, and a leucine structure [19].

The role of WRKY TFs in regulating responses to abiotic stress has been studied in various species. The *IbWRKY47* gene is upregulated in salt stress and confers salinity resistance to sweet potatoes [20]. *MiR156/SPL* induces the expression of the salt-tolerant gene *MdWRKY100*, improving the salt tolerance of *Begonia* [21]. *SbWRKY50* participates in sweet sorghum plant responses to salt stress by controlling its ion balance according to the binding of the upstream promoters of *AtSOS1* and *AtHKT1* [22,23]. In chrysanthemum, *CmWRKY17* is negatively regulated by salt stress [24]. Transcripts, proteins, metabolite levels, hormones, ROS, small RNAs, epigenetic modifications, post-translational modifications, and environmental cues are the main factors regulating the seed germination of *Arabidopsis* and rice [25]. *GhWRKY68* regulates the ABA-mediated pathway and reduce resistance to drought and salt in cotton [26]. The overexpression of the *BdWRKY36* transgenic line can reduce ROS accumulation by the activation of *NtNCED1, NtDREB3,* and *NtLEA5* in the ABA biosynthesis pathway and lead to significant resistance to drought stress in tobacco [27]. In pepper, *CaWRKY6* activates *CaWRKY40* and makes it more resistant to heat and humidity stress [28]. *GmWRKY27* inhibits the *GmNAC29* promoter through an independent inhibitory effect and inhibits the expression of *GmNAC29* with *GmMYB174* to improve plant resistance to salt and drought stress [29]. In pineapple, 54 WRKY genes have been investigated in this family [30]. However, there has been less research regarding functional analyses of the pineapple WRKY family’s genes. Huang *et al.* reported that the ectopic overexpression of *AcWRKY31* negatively affects tolerance to drought and salt stresses in rice [31]. The first-ever study conducted with transgenic pineapple showed the ability of overexpressed *AcWRKY28* in pineapple to confer salt stress tolerance [32]. Through the regulation of bromelain and oxidative stress, the effect of the DA-6 and COS PGRs can create a tolerance to drought in pineapple [33].

Ecological stresses have a significant impact on the fruit development, quality, and yield of pineapple plants [34]. Conventional pineapple breeding for new variety development is time-consuming for commercial production, and self-incompatibility further complicates this process. Molecular breeding and biotechnological tools make breeding programs easier and more efficient. Transgenic *AcWRKY31-OE* pineapple and *Arabidopsis* plants were successfully developed using the *Agrobacterium*-mediated transformation method. The results of this study showed that the overexpression of *AcWRKY31* increased sensitivity to drought and salt tolerance in both pineapple and *Arabidopsis* and increased tolerance to biotic stress caused by pineapple mealybugs in pineapple. In *Arabidopsis*, the overexpression of *AcWRKY31* increased resistance to *Sclerotinia sclerotiorum* infection. The findings of our study on stress-responsive genes provide insight into molecular breeding for developing improved pineapple varieties to boost the economic growth of producers.

## 2. Results

### 2.1. The Expression Profiles and Subcellular Localization of AcWRKY31

**Figure 1:**
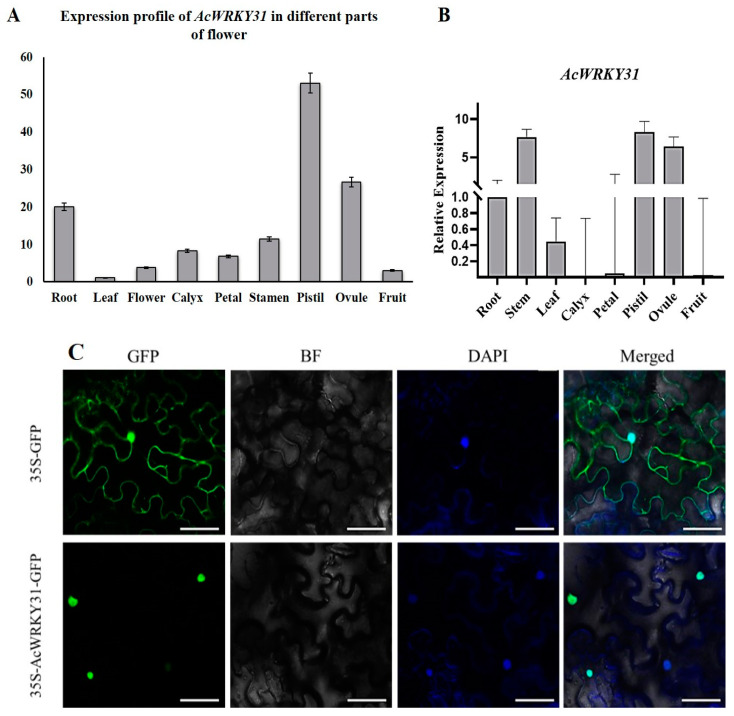
Expression levels of *AcWRKY31* in different parts of pineapple tissues; (**A**) Expression profiles of RNA-sequencing; (**B**) Relative expression levels *AcWRKY31* from RT-qPCR analysis. The error bars indicate ±SD (*n* = 3) (**C**) Subcellular localization of *AcWRKY31* that located in nucleus (Bar = 50 μm; Notes: blue and green color dots indicate the nucleus of cells under microscope).

By analyzing the gene expression patterns, we can preliminarily identify the developmental processes involved and provide a theoretical foundation for subsequent phenotypic and functional analyses. To examine the expression patterns of *AcWRKY31* in different pineapple tissues, we downloaded and analyzed the transcriptome data from various tissues, including root, leaf, flower, calyx, petal, stamen, pistil, ovule, and fruit tissues [35,36]. The expression level of *AcWRKY31* was shown to be the highest in the pistil, followed by the ovule and root (Figure 1A). In addition, an RT-qPCR analysis confirmed the accuracy of the expression level of *AcWRKY31* in different pineapple tissues and revealed high expression levels in the pistil and ovule (Figure 1B). Based on the tobacco transient transformation, we found that *AcWRKY31* is localized in the nucleus (Figure 1C). These expression patterns suggest that *AcWRKY31* serves an important function in pineapple pistil and ovule development as well as in root development for water absorption.

### 2.2. Phylogenetic Analysis and Sequence Alignment of AcWRKY31

**Figure 2:**
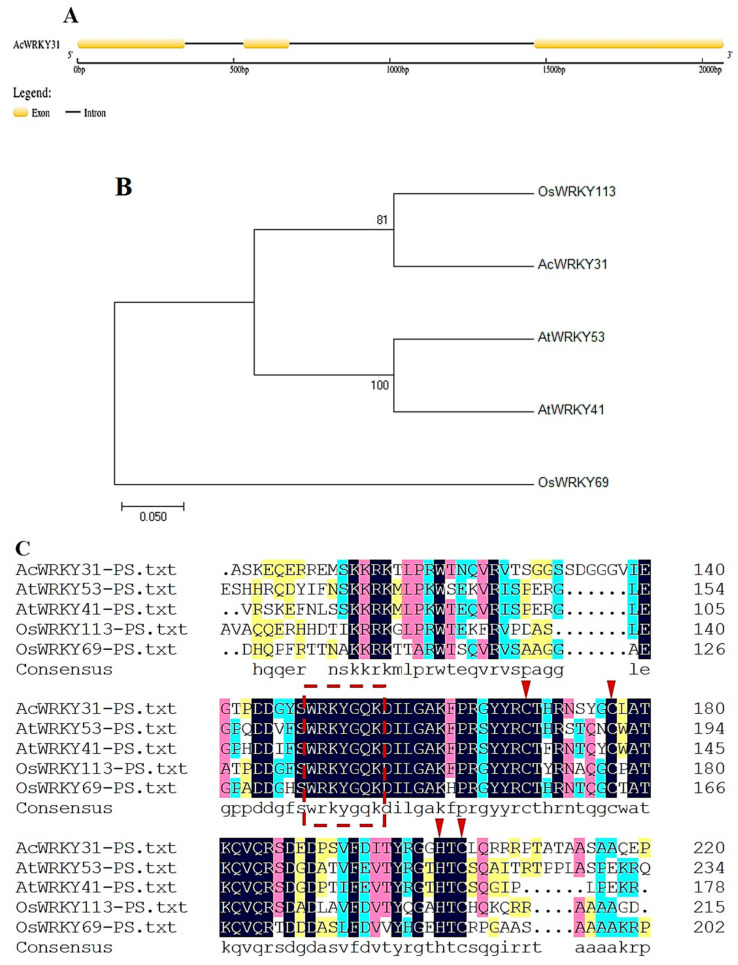
Bioinformatics analysis of *AcWRKY31*. (**A**) Gene structure of *AcWRKY31*. (**B**) Phylogenetic tree of *AcWRKY31* and its homologous genes in *Arabidopsis* and rice. The unrooted phylogenetic tree was generated using MEGA 7.0 software with neighbor-joining procedure and the following parameters: Poisson model, pairwise gap deletion, and 1000 bootstraps. Bar = 0.05 indicates the distance scale. (**C**) Multiple sequence alignment of *AcWRKY31* and its homologous protein sequences (Note: the red dotted line refers to the WRKYGQK domain, and red triangles refer to the C_2_HC-type zinc finger motif).

To characterize the pineapple gene *AcWRKY31*, the CDS sequence and genome sequence were downloaded, and the gene structure is depicted using GSDS 2.0 (Figure 2A). A phylogenetic tree of the pineapple *AcWRKY31* and its homologous genes in *Arabidopsis* and rice was constructed. The phylogenetic tree indicated that *AcWRKY31* shares a high degree of similarity with *AtWRKY53* in *Arabidopsis* and *OsWRKY113* in rice (Figure 2B). In addition, the alignment of multiple protein sequences showed that *AcWRKY31* belongs to group III of the pineapple WRKY TF family, which contains a conserved WRKYGQK domain and C_2_HC-type zinc finger motif (C-X7-C-X23-H-X-C) (Figure 2C).

### 2.3. Transformation and Identification of Overexpressed AcWRKY31 in Pineapple

**Figure 3:**
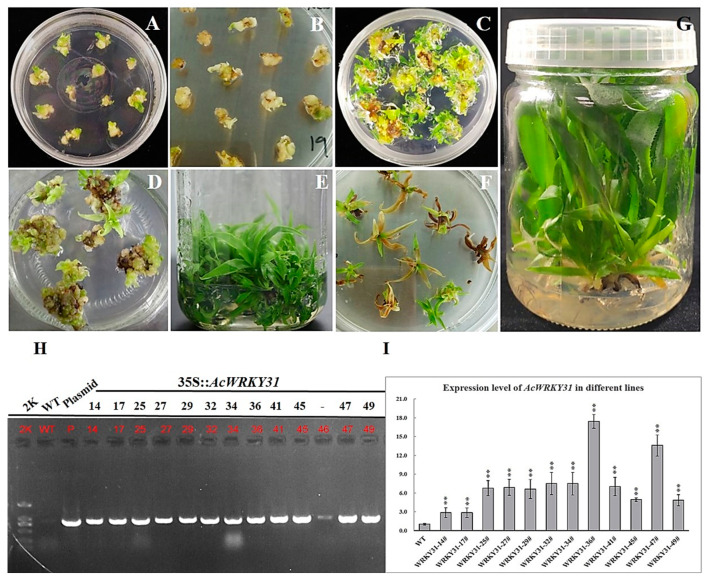
Transformation and identification process of overexpression of *AcWRKY31* in pineapple; (**A**) *Agrobacterium* transformation in pineapple callus; (**B**) Infected callus in 1st selection medium; (**C**) Plant regeneration from resistant callus in normal medium; (**D**) Plant regenerated callus in 2nd selection medium; (**E**) Resistant pineapple plantlets in normal medium; (**F**) Plantlets that died and changed color in 3rd selection medium; (**G**) Completed and resistant pineapple plantlets after undergoing selection three times; (**H**) Confirmation of positive transgenic pineapple by PCR method; (**I**) The expression level of *AcWRKY31-OE* lines and WT pineapple by RT-qPCR analysis. The error bars indicate ±SD (*n* = 3), and the asterisks indicate the significant differences based on Student’s *t*-test (** *p* < 0.01).

To investigate the regulations and functions of *AcWRKY31,* a genetic transformation was performed using an *Agrobacterium*-mediated transformation system as described in Priyadarshani et al. with a few modifications [37]. Transgenic callus and plants were selected rigorously (Figure 3A–G) in the selection medium. The screened plants were further confirmed through PCR testing and sequencing. A total of 12 independent positive *AcWRKY31*-overexpressed pineapple plants were verified (Figure 3H). The WT plants and 12 *AcWRKY31-OE* transgenic pineapple plants were used to extract the total RNA, and RT-qPCR testing was performed to confirm the expression levels of *AcWRKY31*. The result showed that the expression level of *AcWRKY31* was increased more than twice in all of the overexpressed transgenic plants compared to the wild-type plants (Figure 3I).

### 2.4. Overexpression of AcWRKY31 Reduces Tolerance to Salt and Drought Stresses in Pineapple

**Figure 4:**
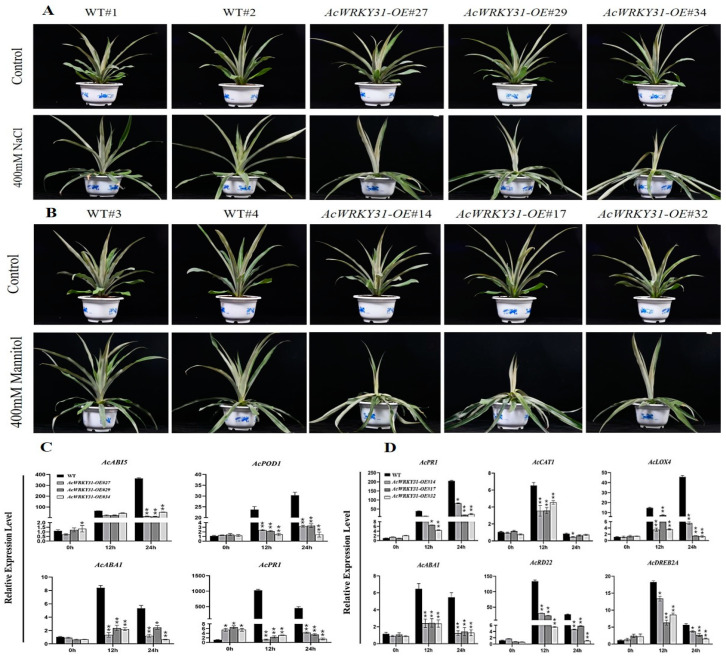
Abiotic stress treatments on transgenic pineapple; (**A**) Comparative phenotypic characters of WT and transgenic pineapple after 3 weeks of salt treatment; (**B**) Comparative phenotypic characters of WT and transgenic pineapple after 3 weeks of mannitol treatment; (**C**,**D**) The expression level of salt- and drought-stress-related genes by RT-qPCR analysis in *AcWRKY31-OE* lines and WT pineapple under abiotic treatments. The error bars indicate ±SD (*n* = 3), and the asterisks indicate significant differences based on Student’s *t*-test (** *p* < 0.01, * *p* < 0.05).

To investigate the function of *AcWRKY31* in pineapple, *AcWRKY31-OE* transgenic plants were subjected to salt and drought stress. After three weeks of treatment with 400 mM NaCl and 400 mM mannitol solutions, the leaves of the *AcWRKY31-OE* pineapple plants were wilted, dried, and drooped, while the leaves of the wild-type (WT) pineapple plants remained upright (Figure 4A,B). After undergoing one month of recovery in a greenhouse, the *AcWRKY31-OE* transgenic pineapple plants were completely dried and noticeably lighter compared to the WT plants. According to the results of the RT-qPCR analysis, enzyme- and salt-stress-related marker genes, such as *AcPOD1*, *AcABI5*, *AcABA1*, and *AcPR1* genes, in the *AcWRKY31-OE* plants showed reduced relative expression levels compared to those of the WT plants (Figure 4C). Similarly, the relative expression levels of hormone- and drought-stress-related marker genes (*AcCAT1*, *AcPR1*, *AcLOX4*, *AcABA1*, *AcRD22*, and *AcDREB2A*) in the *AcWRKY31-OE* plants were lower than then those to the WT pineapple (Figure 4D). These findings indicate the overexpression of *AcWRKY31* enhances the sensitivity to salt and drought stress in pineapple.

### 2.5. Overexpression of AcWRKY31 Increases the Resistance to Pineapple Mealybug

**Figure 5:**
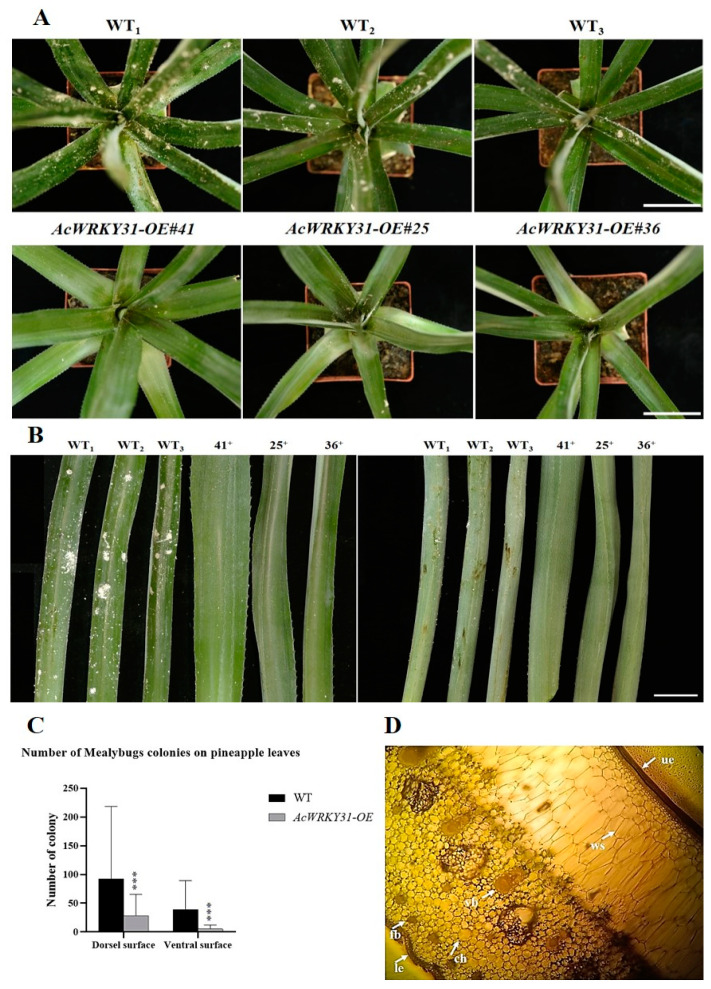
Inoculation of *Dysmicoccus brevipes* (pineapple mealybugs) on pineapple leaves; (**A**) Comparison of infected leaves between WT and *AcWRKY31-OE* pineapple plants after 3 months of inoculation, bars = 1 cm; (**B**) Comparison of infected mealybugs and number of colonies on dorsiventral surfaces in both WT and transgenic leaves (Bar= 1 cm) (**C**) Statistical results of mealybugs’ colonies on dorsiventral surfaces in both WT and transgenic lines (the error bars indicate ±SD (*n* = 3)) (**D**) Free-hand section of pineapple leaves to understand the cell arrangement on both surfaces (ue: upper epidermal layer, le: lower epidermal layer, ch: chlorenchyma cell, ws: water storage cell, vb: vascular bundle, and fb: fiber; Note: cell arrangements are the same in both WT and transgenic lines). The asterisks indicate significant differences (*** *p* < 0.001).

To investigate the function of *AcWRKY31* that response to biotic stress, four-month-old *AcWRKY31-OE* and WT pineapple plants were inoculated with *Dysmicoccus brevipes*. There were no significant differences in *AcWRKY31-OE* and WT plants in the first two months after inoculation. However, after 3 months of inoculation, we observed a significantly higher density of colonies on the crown meristem and dorsal surfaces of the WT leaves compared to that of those the *AcWRKY31-OE* pineapple plants. In addition, the size of the colonies was significantly larger in the WT plants (Figure 5A). In the WT plants, there were approximately 200 colonies on the dorsal surface and less than 100 colonies on the ventral surface of the pineapple leaves, and the smallest colony sizes were dotted while the maximum colony diameter was 0.8 cm. In the transgenic pineapple leaves, the number of mealybug colonies did not exceed 60 on the dorsal surface and was less than 15 on the ventral surfaces. Most of the colonies were tiny, and the largest colonies were only 0.2 cm in diameter (Figure 5B,C). Interestingly, we found that the dorsal surface was more susceptible to mealybug infection in both the WT and transgenic plants compared to the ventral leaf surface. To understand the relationship between the higher infection rate on the dorsal side compared to the ventral side, free-hand transverse sections were cut, and the cellular arrangement was observed under a microscope. We found a soft epidermal cell layer and thick layers of water storage cells on the dorsal side of both the WT and transgenic leaves. On the ventral side of the leaves, rigid epidermal cells, chlorenchyma cells, fibers, and vascular bundles were observed (Figure 5D). According to the cell arrangements in both the WT and transgenic leaves, mealybugs easily penetrate the cell layers and infect the dorsal surfaces rather than the ventral surfaces. Based on the phenotypic characteristics and statistical analysis data of the colonies on the leaf surfaces, we can conclude that the overexpression of *AcWRKY31* enhanced resistance to *Dysmicoccus brevipes* in the pineapple.

### 2.6. RNA-Seq Analysis of AcWRKY31-OE Pineapple

**Figure 6:**
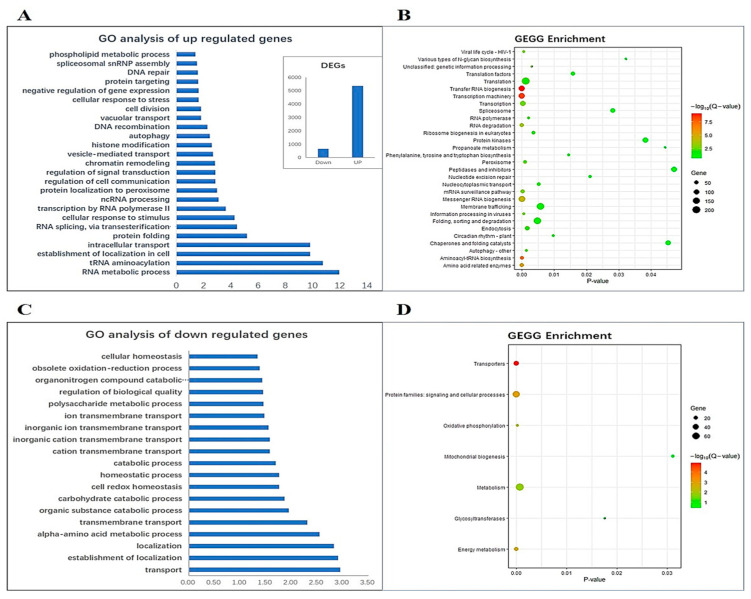
Transcriptomic analysis of WT and *AcWRKY31-OE* transgenic pineapple plants. (**A**) GO analysis of the upregulated genes; (**B**) KEGG pathway analysis for upregulated genes; (**C**) GO analysis of the downregulated genes; (**D**) KEGG pathway analysis for downregulated genes.

To study the functions and mechanisms of *AcWRKY31* regulation in pineapple, a transcriptomic analysis was conducted by using the total RNA from the WT and *AcWRKY31-OE* transgenic pineapple. In this analysis, we identified 5357 upregulated genes and 662 downregulated genes from the transgenic pineapple plants compared to the WT plants. The putative functions of these differentially expressed genes (DEGs) are provided in Supplementary Tables S2.1 and S2.2. The Gene Ontology (GO) enrichment analysis of DEGs showed that 5357 upregulated genes were significantly enriched in the RNA metabolic process, tRNA aminoacylation, the establishment of localization in cell, intracellular transport, protein folding, RNA splicing via transesterification reaction, cellular response to stimulus, transcription according to RNA polymerase II, ncRNA processing, etc. (Figure 6A). The Kyoto Encyclopedia of Genes and Genomes (KEGG) pathway analysis indicated that the upregulated DEGs were mostly involved in the process of translation, protein kinases, peptidases and inhibitors, membrane trafficking, folding, sorting and degradation, endocytosis and translation factors, etc. (Figure 6B). Among the upregulated genes, a total of 30 genes were identified relating to diseases and pathogen response, and further analysis showed that 18 of them contained the conserved WRKY binding domain (W-box TGAC) in the promoter region. These 18 genes typically belong to the CC-NBS-LRR and TIR-NBS-LRR classes of the disease resistance protein family (Supplementary Table S2.3). The GO analysis of 662 downregulated genes revealed their involvement in 19 GO terms, primarily concentrated in transport, the establishment of localization, alpha-amino acid metabolic process, transmembrane transport and the organic substance catabolic process, the carbohydrate catabolic process, etc. (Figure 6C). In addition, the KEGG analysis pointed out that these downregulated genes were activated in seven pathways, especially in mitochondrial biogenesis, metabolism, glycosyltransferases, oxidative phosphorylation, protein families, signaling and cellular processes, energy metabolism, and transporters (Figure 6D). Furthermore, three differentially expressed genes (*Aco010322*: MYB-like transcription factor family protein, *Aco014286*: Disease resistance protein CC-NBS-LRR class family, and *Aco024514*: Tetratricopeptide repeat TPR-like superfamily protein; three that are downregulated (*Aco001317*: Mitochondrial substrate carrier family protein *Aco002224*: Beta-1,4-N-acetylglucosaminyltransferase family protein, and *Aco010847*: Glutaredoxin family protein from downregulated were selected and performed RT-qPCR analysis to confirm the accuracy of the RNA-sequencing data. The results showed that the expression profiles for these six genes are consistent and aligned with the RNA-seq data (Supplementary Figure S3).

### 2.7. AcWRKY31-OE Transgenic Arabidopsis Reduces Tolerance to Salt and Drought Stresses

**Figure 7:**
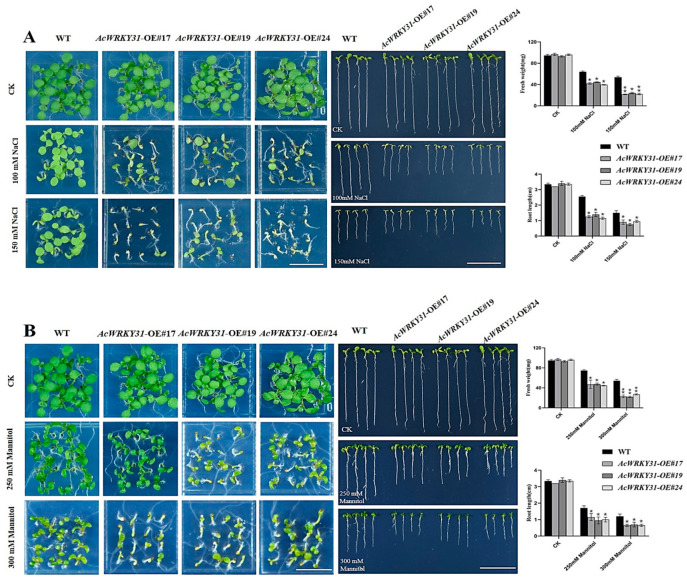

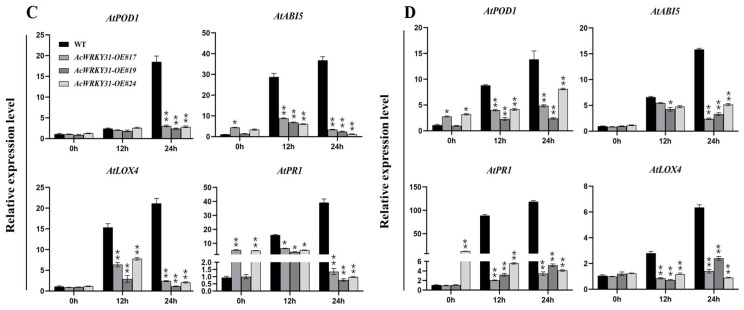
Salt and drought treatments in *AcWRKY31-OE Arabidopsis*; (**A**) Observation of germination rate, fresh weight, and root length of *AcWRKY31-OE* and WT *Arabidopsis* under NaCl treatment (bar = 1 cm); (**B**) Observation of germination rate, fresh weight, and root length of *AcWRKY31-OE* and WT *Arabidopsis* under mannitol treatment (bar = 1 cm); (**C**,**D**) The expression of abiotic-stress-related genes in the WT and *AcWRKY31* transgenic *Arabidopsis* plants in response to salt and drought stresses. Statistical results of fresh weight and root length after 7 days; the error bars indicate ±SD (*n* = 3), and the asterisks indicate the significant differences based on Student’s *t*-test (** *p* < 0.01, * *p* < 0.05).

To confirm the heterologous functions of *AcWRKY31-OE* in *Arabidopsis*, we transformed the overexpressed vector *35S:AcWRKY31-GFP* gene and its homologous gene *35S:AtWRKY53-GFP* into *Arabidopsis* by the floral dip method. The overexpressed *AcWRKY31* and its homologous gene (*AtWRKY53*) in *Arabidopsis* seeds underwent salt and drought treatments. Under the treatment of 100 and 150 mM NaCl, both the WT and transgenic *Arabidopsis* seeds could germinate completely; however, the fresh weight and root length of *AcWRK31-OE Arabidopsis* were significantly reduced compared to the WT plants (Figure 7A). Likewise, a notably adverse impact was observed for the results of the *AcWRK31-OE Arabidopsis* plants subjected to 200 mM and 250 mM mannitol treatments (Figure 7B). Furthermore, the same findings were observed in *AtWRKY53-OE Arabidopsis* under the stress of the salt and drought treatments (Supplementary Figures S4 and S5). Sun and Yu showed that the *Atwrky53* mutant negatively regulates drought tolerance by mediating stomata movement [38]. According to the phenotypic characters of this experiment, *AcWRKY31* plays a negative role in the regulation of drought and salt stress response in *Arabidopsis*. To verify the further molecular mechanism of *AcWRKY31*-*OE Arabidopsis* in salt and drought stress, an RT-qPCR analysis was performed after the treatments. The expression levels of enzyme-related and stress-related genes, such as *AtPOD1, AtLOX4, AtABI5,* and *AtPR1,* were significantly decreased in all of the transgenic lines after the NaCl and mannitol treatments (Figure 7C,D). The results showed that the overexpression of *AcWRKY31* enhanced the sensitivity to salt and drought stress in *Arabidopsis*. Based on the RT-qPCR assay of ABA stress treatment on WT pineapple, we can speculate that *AcWRKY31* may be involved in the ABA-mediated stress response process. To prove this speculation, ABA stress treatments were performed in *AcWRKY31-OE*, *AtWRKY53-OE,* and in *Atwrky53 Arabidopsis* avn showed consistent findings that the ABA was induced in both overexpression lines (Supplementary Figure S6).

### 2.8. AcWRKY31-OE Transgenic Arabidopsis Increases Resistance to the Sclerotinia sclerotiorum Pathogen

**Figure 8:**
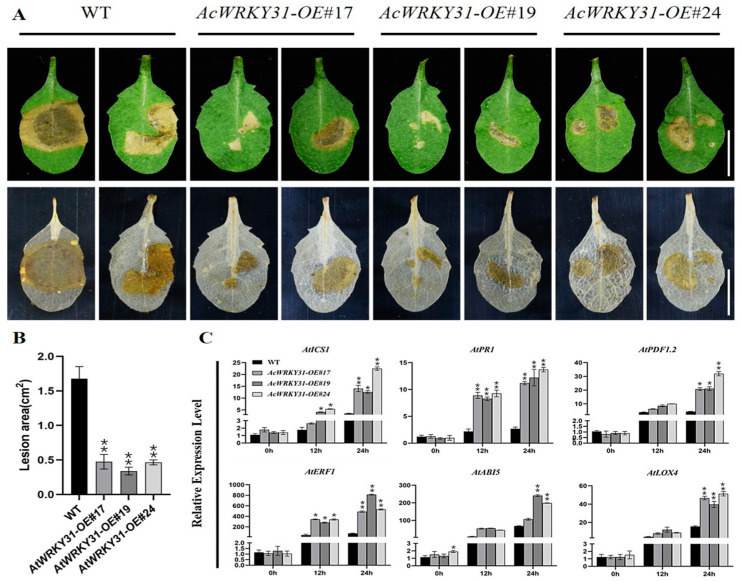
Inoculation of pathogen treatment on *AcWRKY31-OE Arabidopsis.* (**A**) The infection of *Sclerotinia sclerotiorum* on WT and *AcWRKY31-OE Arabidopsis* lines for 24 h and relative plaque area after DAB staining (bar = 1 cm); (**B**) The statistical data of lesion area; (**C**) The expression level of disease-related genes by RT-qPCR in *AcWRKY31-OE* and WT plants. The error bars indicate ±SD (*n* = 3), and the asterisk indicates significant differences based on Student’s *t*-test (** *p* < 0.01, * *p* < 0.05).

**Figure 9:**
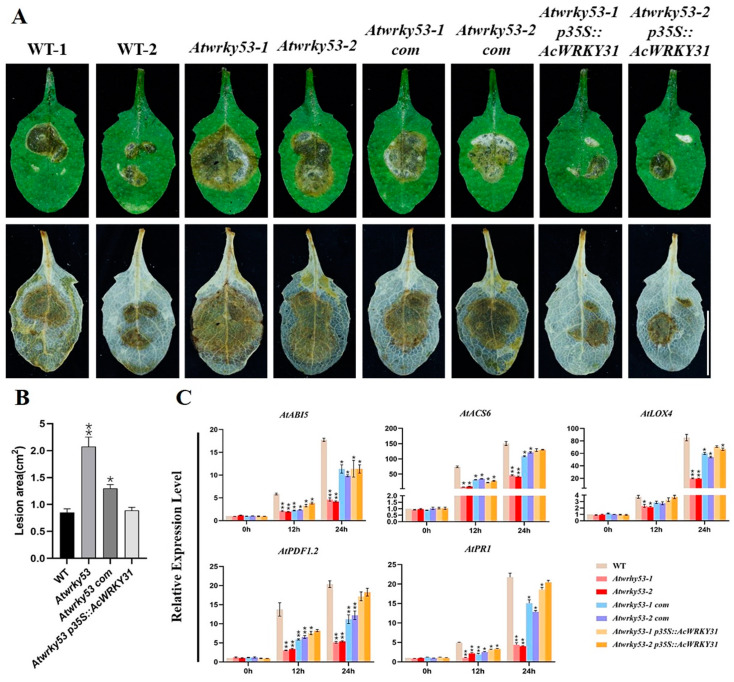
Inoculation of pathogen treatment on *Atwrky53 Arabidopsis.* (**A**) The infection of *Sclerotinia sclerotiorum* on WT, *Atwrky53* mutant, *Atwrky53* complementary lines, and *Atwrky53p35S:AcWRKY31 Arabidopsis* lines for 24 h and relative plaque area after DAB staining (bar = 1 cm); (**B**) The statistical data of lesion area; (**C**) The expression levels of disease-related genes by RT-qPCR in transgenic lines and WT plants. The error bars indicate ±SD (*n* = 3), and the asterisks indicate significant differences based on Student’s *t*-test (** *p* < 0.01, * *p* < 0.05).

To investigate the function of *AcWRKY31* in response to pathogenic infection, leaves of *AcWRKY31-OE* transgenic and WT *Arabidopsis* were inoculated with *Sclerotinia sclerotiorum.* After 24 h of infection, DAB staining was performed to observe the infected area, and the results revealed that the infected area of the WT leaves was significantly larger than that of three *AcWRKY31-OE* lines (Figure 8A,B). Disease- and hormone-related marker genes, such as *AtICS1*, *AtPDF1.2*, *AtPR1*, *AtERF1*, *AtLOX4*, and *AtABI5,* were selected for an RT-qPCR assay after infection [39,40,41]. The RT-qPCR results showed the expression levels of these selected marker genes were significantly increased, indicating that the overexpression of *AcWRKY31* enhanced the plants’ sensitivity to pathogenic disease (Figure 8C). In addition, we inoculated the same pathogen on *Atwrky53*, *Atwrky53-com*, and *Atwrky53pAcWRKY31* leaves. The affected areas of the *Atwrky53pAcWRKY31* leaves were smaller than those of the *Atwrky53* and *Atwrky53-com,* and the expression levels of marker genes were also increased in the *Atwrky53pAcWRKY31* leaves (Figure 9A–C). Therefore, these results provided proof that the functions of *AcWRKY31* and its homologous gene *AtWRKY53* can increase the resistance to biotic stress in both pineapple and *Arabidopsis*.

## 3. Discussion

Environmental factors such as drought, salt, pathogens, and pests have a significant impact on crop yield and quality. Pineapple, an important economic fruit crop, is susceptible to various ecological stresses during its lifespan. WRKY transcription factors (TFs) are involved in biotic and abiotic stress in different crops, such as pepper [42], cotton [43], chrysanthemum [24], and rice [44]. In pineapple, 54 WRKY genes have been identified and reported by the authors of [30,34]. However, only limited research has been conducted to elucidate the functions of WRKY genes in pineapple [31,32]. Therefore, the current study was focused on cloning and transforming the *AcWRKY31* gene to investigate its functions in pineapple.

WRKY transcription factors (TFs) can be divided into three groups (I, II, and III) based on their motifs and domains. Members of group III contain a conserved WRKYGQK domain and a C_2_HC zinc finger structure (C-X7-C-X23-H-X-C). Previous studies have shown that group III WRKY genes in Chinese rose [45], *Sorghum* [46], barley [47], maize [48], rice [49], *Arabidopsis* [50], and pineapple [30] share these same domains and motifs. In our study, *AcWRKY31* exhibited high expression profiles and relative expression levels in the pistils and ovules (Figure 1A,B). The phylogenetic analysis and the alignment of multiple protein sequences revealed that the *AcWRKY31* gene and its homologous genes in rice and *Arabidopsis* belong to the third subfamily from the WRKY transcription factor family, containing a conserved WRKYGQK domain and a C_2_HC zinc finger structure (C-X7-C-X23-H-X-C). Additionally, *AcWRKY31* is exclusively localized in the nucleus and demonstrates transcriptional activation activity (Figure 2). Similarly, our expression profile findings of *AcWRKY31* were consistent with the transcriptomic profile and displayed the same gene structure and functions.

In recent years, abiotic stresses, particularly salt and drought, have severely affected crops [51,52]. Many researchers have investigated and reported on the role of WRKY genes in different crops. For instance, *GhWRKY68* reduces resistance to salt and drought by affecting the germination rate, survival rate, and stomatal closure of transgenic tobacco. [26]. *AtWRKY53* is involved in drought stress and leaf senescence in *Arabidopsis* by mediating stomatal movement and ABA induction [38,42]. In rice, *OsWRKY46*, *OsWRKY64*, and *OsWRKY113* are upregulated in BR IRGA 409 and involve iron toxicity [53]. Our current study demonstrated that *AcWRKY31*-*OE* transgenic pineapples exhibited adverse phenotypic characteristics compared to wild-type pineapples during drought and salt stress. The expression levels of enzyme- and stress-related marker genes, like *AcPOD1*, *AcABI5*, *AcABA1*, and *AcPR1* for salt stress and *AcCAT1*, *AcPR1*, *AcLOX4*, *AcABA1*, *AcRD22*, and *AcDREB2A* for drought stress, were reduced in all transgenic pineapple lines compared to WT plants (Figure 4). In addition, the heterologous overexpression of *AcWRKY31* in *Arabidopsis* also showed an increased sensitivity to salt and drought compared to the WT plants. Therefore, our findings indicate that the overexpression of *AcWRKY31* enhances the sensitivity to abiotic stresses, especially salt and drought, in both pineapple and *Arabidopsis*.

Plants must adapt to drastic environmental changes throughout their lifespan by altering their biochemical and physiological pathways. Among those challenges, attacks from pathogens, insects, pests, and weeds can cause significant damage to plants and reduce crop yields. Many researchers have reported the effects of WRKY TFs on different biotic stresses, for example, the overexpression of *AtWRKY28* conferred resistance to *Sclerotinia* and *Botrytis cinerea* in *Arabidopsis* [54], the overexpression of *AtWRKY75* promoted resistance to *Sclerotinia* and *Pseudomonas syringae* [55], and *AtWRKY70 Arabidopsis* increased the resistance to SA-mediated powdery mildew but rendered plants sensitive to JA-mediated black spot [56]. The *AtWRKY22* genes promote susceptibility to aphids and modulate salicylic acid and jasmonic acid signaling [57]. In pineapple, Zhou et al. stated that *AcWRKY28* mediated the activation of *AcCPK* genes and conferred drought and salt stress tolerance in transgenic pineapple and resistance to *Sclerotinia* in transgenic *Arabidopsis* [32]. In rice, *OsWRKY6* positively regulated resistance to *Xanthomonas oryzae* [58]. The overexpression of *OsWRKY31* and *OsWRKY13* enhanced the resistance to rice blast in rice [59,60]. The pineapple mealybug (*Dysmicoccus brevipes*) is one of the most common insects that affect plant quality and yield by sap-sucking and acts as a vector for the pineapple mealybug wilt-associated virus (PMWaV) [12]. Our result showed that the overexpression of *AcWRKY31* significantly enhances the resistance to mealybugs and *Sclerotinia sclerotiorum* infection. The RT-qPCR analysis of disease-related and hormone-related marker genes (*AtICS1*, *AtPDF1.2*, *AtPR1*, *AtERF1*, *AtLOX4*, and *AtABI5)* also indicated that *AcWRKY31* improved pineapple tolerance to biotic stress.

The WRKY family is also considered to play a crucial role in hormone signaling pathways, including ABA, BRs, ETH, JA, and SA, and can participate in plant defenses [61,62]. The ABA signaling pathway is mainly involved in the regulation of the abiotic stress response in plants [63]. The nuclear protein *GmWRKY12* was responsive to drought, salt, ABA, and salicylic acid (SA) stress [64]. The overexpression of *GhWRKY1* in *Arabidopsis* constitutively activated ABA biosynthesis genes, signaling genes, responsive genes, and drought-related maker genes and led to an enhanced tolerance to drought [65]. Under an exogenous hormone treatment, the expression level of *AcWRKY31* was reduced at 4 h, 8 h, and 12 h, then it instantly increased at 24 h and 48 h under an ABA treatment (Supplementary Figure S1). In addition, the phenotype of *AcWRKY31-OE Arabidopsis* lines in the ABA treatment showed a significantly reduced resistance to ABA than WT plants. During the salt and drought stress experiments, it was found that the expression levels of marker genes in ABA-related signaling pathways were significantly increased. This expression change may be due to the involvement of *AcWRKY31* in the ABA signaling pathway, which coordinates metabolic and physiological processes within plants and leads to changes in gene expression to adapt to alternative environments, but further research is needed. Therefore, we speculate that *AcWRKY31* may regulate plant stress response through ABA-mediated signaling pathways.

**Figure 10:**
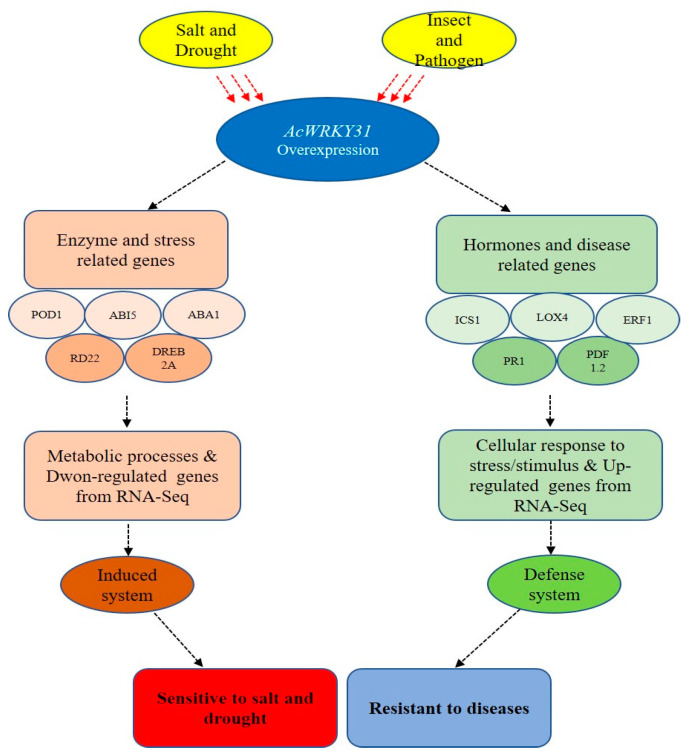
A role model of *AcWRKY31-OE* under biotic and abiotic stresses. *AcWRKY31* gene regulates enzyme- and stress-related genes and then negatively responds to salt and drought stresses while it positively responds to disease by regulating hormone- and disease-related genes.

Altogether, based on transcriptome and experimental results, we conclude that *AcWRKY31* plays distinct roles in pineapple under both biotic and abiotic stress. Its reduction in plant response to drought and salt stress may be mediated by the ABA signaling pathway, while its increase in plant resistance to biological stress may be achieved by regulating the expression of disease-resistance-related genes (Figure 10).

## 4. Materials and Methods

### 4.1. Plant Materials and Pineapple Callus Induction

The pineapple (Tainong 11) plants and *Arabidopsis* (Columbia-0) seeds used in this experiment were obtained from Qin Lab, Center of Genomics Biotechnology, Fujian Agriculture and Forestry University, Fujian, China, where the experiments took place. For pineapple callus induction, we used the pineapple micro-propagation methods of Priyadarshani [66] with some modifications, especially for sterilization processes. Briefly, the explants such as leaf bases and crown meristems were soaked in disinfectant solution overnight to reduce contaminations and then washed with running tap water. In addition, the explants were surface sterilized with 75% ethanol for 1 min and then sterilized with 3–5% sodium hypochlorite solution for 8–10 min. Furthermore, the explants were cut into sizes of 0.5–1.5 cm and then sterilized with 0.2% of HgCl_2_ solution for 10 min. The explants were washed with sterilized double-distilled water 3–5 times after each sterilization. After sterilizations, the samples were air-dried and cultured in full MS solid medium (with pH of −5.8) containing 30% sucrose, 4 mg/L of BAP, and 0.2 mg/L of NAA for callus induction. The callus was subcultured in full MS solid medium containing 1 mg/L of BAP and 0.2 mg/L of NAA. All cultures were kept in a tissue culture room under the following controlled conditions: 8 h/16 h dark and light cycle, light intensity of 3000 lux, and temperature of ±26 °C. All the sterilization and cultural processes were carried out in an aseptic condition.

### 4.2. Vector Construction, Gene Transformation of AcWRKY31, and Transgenic Plant Selection in Pineapple

The peptide sequences of *AcWRKY31* (*Aco000358.1*) and its homologous genes from rice were downloaded from the *Phytozome13* database “https://phytozome-next.jgi.doe.gov/ (accessed on 20 September 2021)” and those of *Arabidopsis* were downloaded from TAIR “https://www.arabidopsis.org/ (accessed on 20 September 2021)”. The CDS sequence (containing 1098 bps) and genome sequence (containing 2068 bps) of *AcWRKY31* were downloaded from the *Phytozome13* database, and the gene structure was displayed by GSDS 2.0. The protein sequences of *AcWRKY31* and its two homologous genes from rice and *Arabidopsis* were used, and the alignment of multiple sequences was carried out using DNAMAN V6.0 software. The specific primers were designed using SnapGene^TM^1.1.3 software. The total RNA from young pineapple leaves was extracted and cloned to the cDNA. The cDNA and gene-specific primers of *AcWRKY31* were used for PCR amplification to obtain the target DNA. After purification of the PCR products, the target DNA was cloned into the pENTER/D-TOPO vector and then recombined into the final pGWB502 vector using LR Clonase II enzyme mix (Thermo Fisher Scientific, Waltham, MA USA). The plasmids from the final vectors were extracted and transformed into *Agrobacterium* (GV3101) strains. The *Agrobacterium* with targeted genes was transformed into pineapple callus. In the transformation process, positive pineapple callus selection and plant screening were carried out as explained by Priyadarshani [37,67] with some modifications, especially in regards to selection time. Time for selection and antibiotic concentration were optimized according to the gene. Three repeated selections were carried out alternatively in the selection and normal medium. Following selection, the resilient plantlets were excised and cultivated on an MS solid medium supplemented with 0.2 mg/L NAA to stimulate root growth. Putative transgenic plants underwent DNA extraction for subsequent PCR amplification. DNA samples yielding positive PCR outcomes were forwarded to Bosun Biotechnology (Shanghai, China) Co., LTD for sequencing.

### 4.3. Analysis of Salt and Drought Stress in AcWRKY31-Overexpressed Pineapple

To analyze the response of transgenic and wild-type (WT) pineapple plants to salt and drought stress, plantlets at comparable growth stages were selected for experimentation. WT and transgenic pineapple plantlets were transplanted into pots with composted soil and placed in a greenhouse at 30 °C ± 2 °C for four months until they grew into adult pineapple plants. After four months, six *AcWRKY31-OE* transgenic plants and WT pineapple plants were treated with 100 mL of 400 mM NaCl solution for salt stress and 400 mM mannitol solution for drought stress. The phenotypic characteristics were noted and recorded daily. In addition, leaf samples of treated WT and *AcWRKY31-OE* transgenic pineapple were collected at 0 h, 12 h, and 24 h, respectively. After irrigating for 3 weeks, the phenotypic characters of treated WT and transgenic pineapple plants were compared. For the molecular assays, the total RNA was isolated from these treated samples and reverse-transcribed into cDNA for further experiments. Selected marker genes were analyzed using RT-qPCR with specific primers (Supplementary Table S1).

### 4.4. Analysis of Biotic Stress in AcWRKY31-OE Transgenic Pineapple

To investigate the role of *AcWRKY31* in biotic stress response, pineapple mealybugs (*Dysmicoccus brevipes*) from a previously infected pineapple plant in the greenhouse were used to inoculate the wild-type (WT) and transgenic pineapple plants. A known number of mealybugs were inoculated onto the leaves of four-month-old, healthy wild-type (WT) and transgenic pineapple plants. Plants were maintained in the greenhouse at 30 °C ± 2 °C and were observed weekly. After four months, disease symptoms, plant growth, and insect colonization were visually inspected and recorded. The statistical data of mealybugs colonies were counted and calculated using IBM SPSS Statistics 24.0.0 software.

### 4.5. Genetic Transformation of AcWRKY31 and AtWRKY53 in Arabidopsis

To confirm and validate the functions of *AcWRKY31*, we transformed *AcWRKY31*, its homologous gene *AtWRKY53*, and knockoff mutant *Atwrky53* into *Arabidopsis* using the floral dip method [68]. Inflorescences were immersed in *Agrobacterium* solution containing the targeted plasmids: p35S:*AcWRKY31* and p35S: *AtWRKY53* [68,69]. Subsequently, the infected plants were allowed to grow until the seeds were ready for harvesting. The seeds from the T_0_ generation were germinated in soil and selected by spraying them with Basta (1:1000 dilution of water) to obtain transgenic plants. DNA was extracted from selected putative plants by using the CTAB method [70] to identify the positive plants for T_1_ generation through PCR. The second-generation seeds were obtained by repeated screening and identification, and they were used for subsequent studies.

### 4.6. Analysis of Abiotic Treatments in Transgenic Arabidopsis

The previous study reported that the expression of WRKY TFs in pineapple can be affected by temperature stresses (4 °C and 45 °C), drought, and salt stresses [31]. To understand the regulation of heterologous overexpression of *AcWRKY31* under abiotic stress, the wild-type and T_2_ generation of transgenic *Arabidopsis* seeds were treated with 100 mM–150 mM of NaCl for salt stress, 250 mM–300 mM of mannitol for drought stress, and 0.25–0.75 µM of ABA for ABA stress. After 7 days, the germination rates, fresh weight, and root length were investigated. To analyze the expression level of *AcWRKY31*, RT-qPCR analysis was performed for salt and drought stress with stress-related marker genes. All the experiments were conducted with three replicates.

### 4.7. Analysis of Sclerotinia sclerotiorum Inoculation on Transgenic Arabidopsis

To examine the functions of *AcWRKY31* in response to pathogens, WT and three independent lines of *AcWRKY31-OE*, *AtWRKY53-OE*, *Atwrky53*, *Atwrky53-com,* and *Atwrky53-AcWRKY31* T_2_-generation *Arabidopsis* seeds were used. The preserved fungal strain of *Sclerotinia sclerotiorum* was sub-cultured on potato dextrose agar medium for the production of new hyphae. Briefly, five seeds from each line were planted in plastic pots with composted soil for germination. Leaves from 3-week-old transgenic and WT *Arabidopsis* plants were inoculated with the same quantity of *Sclerotinia sclerotiorum.* The infected area was analyzed after 24 h with DAB staining to analyze the accumulation of reactive oxygen species. For DAB staining, leaves were immersed in 50 mL of DAB aqueous solution (0.05 g DAB; ddH_2_O 50 mL) overnight in darkness. Dyed leaves were de-colorized using 75% ethanol in a water bath for 15 min. The lysis areas were recorded by taking photographs and measured using ImageJ 1.51j/Java 1.8.0-112(64-bit) software [71]. The data were analyzed using the Student’s *t*-test and GraphPad Prism 8.3.0 (538). All the experiments were repeated three times.

### 4.8. Analysis of Transcriptomic Data

Total RNA was extracted from three biological replicates of wild-type (WT) and *AcWRKY31*-overexpressing (*AcWRKY31-OE*) pineapple leaves using the Trizol method (Invitrogen, Carlsbad, CA, USA) following the manufacturer’s instructions. Raw reads and adapter sequences were processed to remove ambiguities and adapters using TRIMMOMATIC v 0.3 software [72]. The RNA libraries were sequenced by the Novogene company on a HiSeq2500 sequencing instrument using 150 bp paired-end protocols. The processed reads were aligned to the reference genome using Tophat v2.1.1 software with default parameters. Then, the transcripts were quantified and assembled according to Cufflinks, and the differently expressed genes (DEGs) were identified. Differential expression was determined based on a log2 fold change −1≤ and ≥+1 and a value of FDR ≤ 0.05) [73]. Kyoto Encyclopedia of Genomes and Genes (KEGG) and Gene Ontology (GO) analyses of DEGs were carried out using TBtools v1.09 software [74] and R package UpSet v1.0.0. The promoter region analysis was performed through the online PlantCARE database (http://bioinformatics.psb.ugent.be/webtools/plantcare/html/) (accessed on 13 March 2024). RT-qPCR analysis of six selected genes from up- and downregulated genes was performed to actuate the function of *AcWRKY31* with the RNA-Seq data. The raw data sequence in Fastq format was deposited in the China National Center for Bioinformation Database (https://bigd.big.ac.cn; BioProject ID: PRJCA019756) (accessed on 13 September 2023).

### 4.9. Quantitative Real-Time PCR Analysis

The cDNA was synthesized from extracted total RNA according to the instructions of AMV reverse-transcriptase kit (Takara, Shiga, Japan) [75]. RT-qPCR was carried out using SYBR Premix Ex Taq II (Takara, Japan) system and Bio-Rad Real-Time PCR system. The reaction was conducted in a total volume of 20 μL with 10 μL of enzyme mix, 7.5 μL of nuclease-free water, 1.5 μL of cDNA, and 0.5 μL of each specific forward and reverse primer. The RT-qPCR was performed by using given conditions (95 °C—30 s, 95 °C—5 s, 60 °C—34 s, and 95 °C—15 s) with 40 cycles [76]. The experiment was performed in triplicate. According to the comparative threshold period (2^−ΔΔCt^) protocol, the level of relative expression in stress-related genes was analyzed and confirmed [77].

## 5. Conclusions

The outcomes of our current research highlight the notable expression patterns of *AcWRKY31*, particularly in the pistil and ovule of pineapple. Notably, our investigations reveal that the upregulation of *AcWRKY31* in both pineapple and *Arabidopsis* plants is correlated with a reduced tolerance to salt and drought stress, alongside a heightened resistance to pests and pathogens. These findings underscore the intricate response mechanisms of pineapple to diverse stressors. Consequently, our study yields significant insights crucial for the development of advanced biotechnological interventions and breeding approaches aimed at enhancing the resilience and productivity of pineapple varieties.

## Data Availability

All analytical data of this study are described in the article and its additional files.

## Supplementary Materials

The following supporting information can be downloaded at: https://www.mdpi.com/article/10.3390/plants13131850/s1. Figure S1: Relative expression levels of *AcWRKY31* in different hormone treatments (A) Abscisic Acid (ABA) (B) Ethylene (ETH) (C) Jasmonate Acid (JA) and (D) Salicylic Acid (SA). Figure S2: Analysis of transcriptional activation activity of *AcWRKY31* (A) The growth of *AcWRKY31*-BD/AD, negative and positive control yeast on SD-Leu-Trp. (B) The growth of *AcWRKY31*-BD/AD and negative-positive co-transformed yeast on SD-Leu-Trp-Ade-His+X-α-gal medium. Figure S3: Relative expression levels of Up Regulated Genes and Down Regulated Genes from RNA sequence analysis of WT and transgenic pineapples lines, the error bars indicate ±SD (*n* = 3). Figure S4: Salt treatment on *AcWRKY53-OE Arabidopsis* (A) Observation of germination rate and root length, bar = 1 cm (B) Statistical results of fresh weight and root length of *AcWRKY53-OE* and WT *Arabidopsis* after 7 days of NaCl treatment, the error bars indicate ±SD (*n* = 3) the asterisks indicate the different significances based on Student’s *t*-test (** *p* < 0.01, * *p* < 0.05). Figure S5: Drought treatment on *AcWRKY53-OE Arabidopsis* (A) Observation of germination rate and root length, bar = 1 cm (B) Statistical results of fresh weight and root length of *AcWRKY53-OE* and WT *Arabidopsis* after 7 days of Mannitol treatment, the error bars indicate ±SD (*n* = 3) the asterisks indicate the different significances based on Student’s *t*-test (** *p* < 0.01, * *p* < 0.05). Figure S6: ABA treatment on *Arabidopsis* (A) Observation of germination rate and root length of *AcWRKY53-OE* and WT *Arabidopsis* (bar = 1 cm) (B) Observation of germination rate and root length of *AtWRKY53* mutant, complementary and WT *Arabidopsis* (bar = 1 cm); Statistical results of fresh weight and root length after 7 days (Notes: the error bars indicate ±SD (*n* = 3) the asterisks indicate the different significances based on Student’s *t*-test (** *p* < 0.01, * *p* < 0.05)). Table S1: Primer sequences. Table S2: Up and down- regulated Differential Expressed Genes.

## Abbreviations

WT	Wild-type
TFs	Transcription factors
OE	Overexpression
DEGs	Differentially expressed genes
PCR	Polymerase chain reaction
RT-qPCR	Quantitative real-time polymerase chain reaction
PGRs	Plant growth regulators
DA-6	Diethyl aminoethyl hexanoate
COS	Chitosan oligosaccharide
ABA	Abscisic acid
BRs	Brassinosteroids
ETH	Ethylene
JA	Jasmonate acid
SA	Salicylic acid
MS	Murishige and Skoog medium
BAP	6-Benzylaminopurine
NAA	Naphthalene acetic acid
GFP	Green fluorescent protein
DNA	Deoxyribonucleic acid
cDNA	Complementary DNA
NaCl	Sodium chloride
ROS	Relative oxygen species
Cotton:	
*GhHBA*	High level of beta-amylase activity
Tobacco:	
*NtNCED1*	9-Cis-Epoxycarotenoid Dioxygenase 1
*NtDREB3*	Dehydration Response Element-Binding protein 3
*NtLEA5*	Late Embryogenesis Abundant-like 5
Pineapple:	
*AcPOD*	Peroxidase
*AcABI5*	ABA Insensitive 5
*AcABA1*	ABA Deficient 1
*AcPR1*	Pathogenesis-Related Gene 1
*AcCAT1*	Catalase 1
*AcLOX4*	Lipoxygenase 4
*AcRD22*	Responsive to Desiccation 22
*AcDREB2A*	Dehydration Response Element-Binding protein 2
*AcCPK*	Calcium-dependent Protein Kinase
*Arabidopsis*:	
*AtSOS1*	Salt Overly Sensitive 1
*AtHKT1*	High-Affinity K+ Transporter 1
*AtICS1*	Isochorismate Synthase 1 (SA related)
*AtPDF1.2*	Plant Defensin 1.2
*AtPR1*	Pathogenesis-Related Gene 1
*AtLOX4*	Lipoxygenase 4 (JA related)
*AtERF1*	Ethylene Response Factor 1
*AtABI5*	ABA Insensitive 5

## References

[1] Sinha A.K., Jaggi M., Raghuram B., Tuteja N. (2011). Mitogen-activated protein kinase signaling in plants under abiotic stress. Plant Signal. Behav..

[2] Rodziewicz P., Swarcewicz B., Chmielewska K., Wojakowska A., Stobiecki M. (2013). Influence of abiotic stresses on plant proteome and metabolome changes. Acta Physiol. Plant..

[3] Gong Z., Xiong L., Shi H., Yang S., Herrera-Estrella L.R., Xu G., Chao D.Y., Li J., Wang P.Y., Qin F. (2020). Plant abiotic stress response and nutrient use efficiency. Sci. China Life Sci..

[4] Boisson-Dernier A., Roy S., Kritsas K., Grobei M.A., Jaciubek M., Schroeder J.I., Grossniklaus U. (2009). Disruption of the pollen-expressed FERONIA homologs ANXUR1 and ANXUR2 triggers pollen tube discharge. Development.

[5] Arif Y., Singh P., Siddiqui H., Bajguz A., Hayat S. (2020). Salinity induced physiological and biochemical changes in plants: An omic approach towards salt stress tolerance. Plant Physiol. Biochem..

[6] Shao H.B., Chu L.Y., Shao M.A., Jaleel C.A., Mi H.M. (2008). Higher plant antioxidants and redox signaling under environmental stresses. Comptes Rendus Biol..

[7] Gu R., Song X.F., Liu X.F., Yan L.Y., Zhou Z.Y., Zhang X.L. (2020). Genome-wide analysis of CsWOX transcription factor gene family in cucumber (*Cucumis sativus* L.). Sci. Rep..

[8] Evans M., Kermicle J.L. (2001). Interaction between maternal effect and zygotic effect mutations during maize seed development. Genetics.

[9] Ranjan A., Westrick N.M., Jain S., Piotrowski J.S., Ranjan M., Kessens R., Stiegman L., Grau C.R., Conley S.P., Smith D.L. (2019). Resistance against Sclerotinia sclerotiorum in soybeans involves reprogramming of the phenylpropanoid pathway and up-regulation of antifungal activity targeting ergosterol biosynthesis. Plant Biotechnol. J..

[10] Caceres M., Hidalgo W., Stashenko E., Torres R., Ortiz C. (2020). Essential Oils of Aromatic Plants with Antibacterial, Anti-Biofilm and Anti-Quorum Sensing Activities against Pathogenic Bacteria. Antibiotics.

[11] Santa-Cecília L.V.C., Prado E., Souza B. (2016). Probing behavior of Dysmicoccus brevipes mealybug in pineapple plants1. Pesqui. Agropecu. Trop..

[12] Rohrbach K.G., Johnson M.W. (2003). Pests, diseases and weeds. The Pineapple: Botany, Production and Uses.

[13] Bolton M.D., Thomma B.P., Nelson B.D. (2006). *Sclerotinia sclerotiorum* (Lib.) de Bary: Biology and molecular traits of a cosmopolitan pathogen. Mol. Plant Pathol..

[14] Zhou F., Zhang X.L., Li J.L., Zhu F.X. (2014). Dimethachlon Resistance in *Sclerotinia sclerotiorum* in China. Plant Dis..

[15] Phukan U.J., Jeena G.S., Shukla R.K. (2016). WRKY Transcription Factors: Molecular Regulation and Stress Responses in Plants. Front. Plant Sci..

[16] Xu Y.H., Sun P.W., Tang X.L., Gao Z.H., Zhang Z., Wei J.H. (2020). Genome-wide analysis of WRKY transcription factors in *Aquilaria sinensis* (Lour.) Gilg. Sci. Rep..

[17] Xie Z., Zhang Z.L., Zou X., Huang J., Ruas P., Thompson D., Shen Q.J. (2005). Annotations and functional analyses of the rice WRKY gene superfamily reveal positive and negative regulators of abscisic acid signaling in aleurone cells. Plant Physiol..

[18] Rushton P.J., Bokowiec M.T., Han S., Zhang H., Brannock J.F., Chen X., Laudeman T.W., Timko M.P. (2008). Tobacco transcription factors: Novel insights into transcriptional regulation in the Solanaceae. Plant Physiol..

[19] Chen L., Song Y., Li S., Zhang L., Zou C., Yu D. (2012). The role of WRKY transcription factors in plant abiotic stresses. Biochim. Biophys. Acta.

[20] Chang X., Yang Z., Zhang X., Zhang F., Huang X., Han X. (2022). Transcriptome-wide identification of WRKY transcription factors and their expression profiles under different stress in *Cynanchum thesioides*. PeerJ.

[21] Ma Y., Xue H., Zhang F., Jiang Q., Yang S., Yue P., Wang F., Zhang Y., Li L., He P. (2021). The miR156/SPL module regulates apple salt stress tolerance by activating MdWRKY100 expression. Plant Biotechnol. J..

[22] Chen C., Shang X., Sun M., Tang S., Khan A., Zhang D., Yan H., Jiang Y., Yu F., Wu Y. (2022). Comparative Transcriptome Analysis of Two Sweet Sorghum Genotypes with Different Salt Tolerance Abilities to Reveal the Mechanism of Salt Tolerance. Int. J. Mol. Sci..

[23] Song Y., Li J., Sui Y., Han G., Zhang Y., Guo S., Sui N. (2020). The sweet sorghum SbWRKY50 is negatively involved in salt response by regulating ion homeostasis. Plant Mol. Biol..

[24] Li P., Song A., Gao C., Wang L., Wang Y., Sun J., Jiang J., Chen F., Chen S. (2015). Chrysanthemum WRKY gene CmWRKY17 negatively regulates salt stress tolerance in transgenic chrysanthemum and Arabidopsis plants. Plant Cell Rep..

[25] Zhao J., He Y., Zhang H., Wang Z. (2024). Advances in the molecular regulation of seed germination in plants. Seed Biol..

[26] Jia H., Wang C., Wang F., Liu S., Li G., Guo X. (2015). GhWRKY68 reduces resistance to salt and drought in transgenic *Nicotiana benthamiana*. PLoS ONE.

[27] Sun J., Hu W., Zhou R., Wang L., Wang X., Wang Q., Feng Z., Li Y., Qiu D., He G. (2015). The *Brachypodium distachyon* BdWRKY36 gene confers tolerance to drought stress in transgenic tobacco plants. Plant Cell Rep..

[28] Cai H., Yang S., Yan Y., Xiao Z., Cheng J., Wu J., Qiu A., Lai Y., Mou S., Guan D. (2015). CaWRKY6 transcriptionally activates CaWRKY40, regulates *Ralstonia solanacearum* resistance, and confers high-temperature and high-humidity tolerance in pepper. J. Exp. Bot..

[29] Wang F., Chen H.W., Li Q.T., Wei W., Li W., Zhang W.K., Ma B., Bi Y.D., Lai Y.C., Liu X.L. (2015). GmWRKY27 interacts with GmMYB174 to reduce the expression of GmNAC29 for stress tolerance in soybean plants. Plant J..

[30] Xie T., Chen C., Li C., Liu J., Liu C., He Y. (2018). Genome-wide investigation of WRKY gene family in pineapple: Evolution and expression profiles during development and stress. BMC Genom..

[31] Huang Y., Chen F., Chai M., Xi X., Zhu W., Qi J., Liu K., Ma S., Su H., Tian Y. (2022). Ectopic Overexpression of Pineapple Transcription Factor AcWRKY31 Reduces Drought and Salt Tolerance in Rice and Arabidopsis. Int. J. Mol. Sci..

[32] Zhou Q., Priyadarshani S.V.G.N., Qin R., Cheng H., Luo T., Wai M.H., Mohammadi M.A., Liu Y., Liu C., Cai H. (2024). AcWRKY28-mediated activation of AcCPK genes confers salt tolerance in pineapple (*Ananas comosus*). Hortic. Plant J..

[33] Huang X., Rao G., Peng X., Xue Y., Hu H., Feng N., Zheng D. (2023). Effect of plant growth regulators DA-6 and COS on drought tolerance of pineapple through bromelain and oxidative stress. BMC Plant Biol..

[34] Ming R., Wai C.M., Guyot R. (2016). Pineapple Genome: A Reference for Monocots and CAM Photosynthesis. Trends Genet..

[35] Gao Y., Yao Y., Chen X., Wu J., Wu Q., Liu S., Guo A., Zhang X. (2022). Metabolomic and transcriptomic analyses reveal the mechanism of sweet-acidic taste formation during pineapple fruit development. Front. Plant Sci..

[36] Xie T., Zhang J., Luan A., Zhang W., Wu J., Cai Z., He Y. (2021). Comparative transcriptome analysis of a fan-shaped inflorescence in pineapple using RNA-seq. Genomics.

[37] Priyadarshani S., Cai H., Zhou Q., Liu Y., Cheng Y., Xiong J., Patson D.L., Cao S., Zhao H., Qin Y. (2019). An Efficient Agrobacterium Mediated Transformation of Pineapple with GFP-Tagged Protein Allows Easy, Non-Destructive Screening of Transgenic Pineapple Plants. Biomolecules.

[38] Sun Y., Yu D. (2015). Activated expression of AtWRKY53 negatively regulates drought tolerance by mediating stomatal movement. Plant Cell Rep..

[39] Cai J., Liu T., Li Y., Ow D.W. (2021). A C-terminal fragment of Arabidopsis OXIDATIVE STRESS 2 can play a positive role in salt tolerance. Biochem. Biophys. Res. Commun..

[40] Xiang S., Wu S., Zhang H., Mou M., Chen Y., Li D., Wang H., Chen L., Yu D. (2020). The PIFs Redundantly Control Plant Defense Response against Botrytis cinerea in Arabidopsis. Plants.

[41] Grebner W., Stingl N.E., Oenel A., Mueller M.J., Berger S. (2013). Lipoxygenase6-dependent oxylipin synthesis in roots is required for abiotic and biotic stress resistance of Arabidopsis. Plant Physiol..

[42] Zentgraf U., Laun T., Miao Y. (2010). The complex regulation of WRKY53 during leaf senescence of Arabidopsis thaliana. Eur. J. Cell Biol..

[43] Ren X., Chen Z., Liu Y., Zhang H., Zhang M., Liu Q., Hong X., Zhu J.K., Gong Z. (2010). ABO3, a WRKY transcription factor, mediates plant responses to abscisic acid and drought tolerance in Arabidopsis. Plant J..

[44] Jaglo-Ottosen K.R., Gilmour S.J., Zarka D.G., Schabenberger O., Thomashow M.F. (1998). Arabidopsis CBF1 overexpression induces COR genes and enhances freezing tolerance. Science.

[45] Yan X., Zhao J., Huang W., Liu C., Hao X., Gao C., Deng M., Wen J. (2024). Genome-Wide Identification of WRKY Transcription Factor Family in Chinese Rose and Response to Drought, Heat, and Salt Stress. Genes.

[46] Baillo E.H., Hanif M.S., Guo Y., Zhang Z., Xu P., Algam S.A. (2020). Genome-wide Identification of WRKY transcription factor family members in sorghum (*Sorghum bicolor* (L.) Moench). PLoS ONE.

[47] Zheng J., Zhang Z., Tong T., Fang Y., Zhang X., Niu C., Li J., Wu Y., Xue D., Zhang X. (2021). Genome-Wide Identification of WRKY Gene Family and Expression Analysis under Abiotic Stress in Barley. Agronomy.

[48] Wei K.F., Chen J., Chen Y.F., Wu L.J., Xie D.X. (2012). Molecular phylogenetic and expression analysis of the complete WRKY transcription factor family in maize. DNA Res..

[49] Jeyasri R., Muthuramalingam P., Satish L., Adarshan S., Lakshmi M.A., Pandian S.K., Chen J.-T., Ahmar S., Wang X., Mora-Poblete F. (2021). The Role of OsWRKY Genes in Rice When Faced with Single and Multiple Abiotic Stresses. Agronomy.

[50] Song Y., Gao J. (2014). Genome-wide analysis of WRKY gene family in Arabidopsis lyrata and comparison with *Arabidopsis thaliana* and *Populus trichocarpa*. Chin. Sci. Bull..

[51] Fei X., Hou L., Shi J., Yang T., Liu Y., Wei A. (2018). Patterns of Drought Response of 38 WRKY Transcription Factors of *Zanthoxylum bungeanum* Maxim. Int. J. Mol. Sci..

[52] Cheng Y., Wang Y., Sun J., Liao Z., Ye K., Hu B., Dong C., Li Z., Deng F., Wang L. (2023). Unveiling the genomic blueprint of salt stress: Insights from *Ipomoea pes-caprae* L.. Seed Biol..

[53] Viana V.E., Marini N., Finatto T., Ezquer I., Busanello C., Dos Santos R.S., Pegoraro C., Colombo L., Costa de Oliveira A. (2017). Iron excess in rice: From phenotypic changes to functional genomics of WRKY transcription factors. Genet. Mol. Res..

[54] Windram O., Madhou P., McHattie S., Hill C., Hickman R., Cooke E., Jenkins D.J., Penfold C.A., Baxter L., Breeze E. (2012). Arabidopsis defense against *Botrytis cinerea*: Chronology and regulation deciphered by high-resolution temporal transcriptomic analysis. Plant Cell.

[55] Encinas-Villarejo S., Maldonado A.M., Amil-Ruiz F., de los Santos B., Romero F., Pliego-Alfaro F., Munoz-Blanco J., Caballero J.L. (2009). Evidence for a positive regulatory role of strawberry (*Fragaria* × *ananassa*) Fa WRKY1 and Arabidopsis At WRKY75 proteins in resistance. J. Exp. Bot..

[56] Li J., Brader G., Kariola T., Palva E.T. (2006). WRKY70 modulates the selection of signaling pathways in plant defense. Plant J..

[57] Kloth K.J., Wiegers G.L., Busscher-Lange J., van Haarst J.C., Kruijer W., Bouwmeester H.J., Dicke M., Jongsma M.A. (2016). AtWRKY22 promotes susceptibility to aphids and modulates salicylic acid and jasmonic acid signaling. J. Exp. Bot..

[58] Hwang S.H., Yie S.W., Hwang D.J. (2011). Heterologous expression of OsWRKY6 gene in *Arabidopsis* activates the expression of defense-related genes and enhances resistance to pathogens. Plant Sci..

[59] Zhang J., Peng Y., Guo Z. (2008). Constitutive expression of pathogen-inducible OsWRKY31 enhances disease resistance and affects root growth and auxin response in transgenic rice plants. Cell Res..

[60] Qiu D., Xiao J., Xie W., Liu H., Li X., Xiong L., Wang S. (2008). Rice Gene Network Inferred from Expression Profiling of Plants Overexpressing OsWRKY13, a Positive Regulator of Disease Resistance. Mol. Plant.

[61] Dietz K.J., Vogel M.O., Viehhauser A. (2010). AP2/EREBP transcription factors are part of gene regulatory networks and integrate metabolic, hormonal, and environmental signals in stress acclimation and retrograde signaling. Protoplasma.

[62] Liu Z.Q., Yan L., Wu Z., Mei C., Lu K., Yu Y.T., Liang S., Zhang X.F., Wang X.F., Zhang D.P. (2012). Cooperation of three WRKY-domain transcription factors WRKY18, WRKY40, and WRKY60 in repressing two ABA-responsive genes ABI4 and ABI5 in Arabidopsis. J. Exp. Bot..

[63] Khoso M.A., Hussain A., Ritonga F.N., Ali Q., Channa M.M., Alshegaihi R.M., Meng Q., Ali M., Zaman W., Brohi R.D. (2022). WRKY transcription factors (TFs): Molecular switches to regulate drought, temperature, and salinity stresses in plants. Front. Plant Sci..

[64] Shi W.Y., Du Y.T., Ma J., Min D.H., Jin L.G., Chen J., Chen M., Zhou Y.B., Ma Y.Z., Xu Z.S. (2018). The WRKY Transcription Factor GmWRKY12 Confers Drought and Salt Tolerance in Soybean. Int. J. Mol. Sci..

[65] Hu Q., Ao C., Wang X., Wu Y., Du X. (2021). GhWRKY1-like, a WRKY transcription factor, mediates drought tolerance in *Arabidopsis* via modulating ABA biosynthesis. BMC Plant Biol..

[66] Priyadarshani S.V.G.N., Hu B., Li W., Ali H., Jia H., Zhao L., Ojolo S.P., Azam S.M., Xiong J., Yan M. (2018). Simple protoplast isolation system for gene expression and protein interaction studies in pineapple (*Ananas comosus* L.). Plant Methods.

[67] He Y., Luan A., Wu J., Zhang W., Lin W. (2023). Overcoming key technical challenges in the genetic transformation of pineapple. Trop. Plants.

[68] Zhang X., Henriques R., Lin S.S., Niu Q.W., Chua N.H. (2006). Agrobacterium-mediated transformation of *Arabidopsis thaliana* using the floral dip method. Nat. Protoc..

[69] Clough S.J., Bent A.F. (1998). Floral dip a simplified method for Agrobacterium-mediated transformation of *Arabidopsis thaliana*. Plant J..

[70] Aydemir B.C., Ozmen C.Y., Kibar U., Mutaf F., Buyuk P.B., Bakir M., Ergul A. (2020). Salt stress induces endoplasmic reticulum stress-responsive genes in a grapevine rootstock. PLoS ONE.

[71] Bowler C., Benvenuto G., Laflamme P., Molino D., Probst A.V., Tariq M., Paszkowski J. (2004). Chromatin techniques for plant cells. Plant J..

[72] Bolger A.M., Lohse M., Usadel B. (2014). Trimmomatic: A flexible trimmer for Illumina sequence data. Bioinformatics.

[73] Trapnell C., Roberts A., Goff L., Pertea G., Kim D., Kelley D.R., Pimentel H., Salzberg S.L., Rinn J.L., Pachter L. (2012). Differential gene and transcript expression analysis of RNA-seq experiments with TopHat and Cufflinks. Nat. Protoc..

[74] Chen C., Chen H., Zhang Y., Thomas H.R., Frank M.H., He Y., Xia R. (2020). TBtools: An Integrative Toolkit Developed for Interactive Analyses of Big Biological Data. Mol. Plant.

[75] Roth R., Madhani H.D., Garcia J.F. (2018). Total RNA Isolation and Quantification of Specific RNAs in Fission Yeast. Methods Mol. Biol..

[76] Singh M., Goel S., Meeley R.B., Dantec C., Parrinello H., Michaud C., Leblanc O., Grimanelli D. (2011). Production of Viable Gametes without Meiosis in Maize Deficient for an ARGONAUTE Protein. Plant Cell.

[77] Sang J., Han X., Liu M., Qiao G., Jiang J., Zhuo R. (2013). Selection and validation of reference genes for real-time quantitative PCR in hyperaccumulating ecotype of Sedum alfredo under different heavy metals stresses. PLoS ONE.

