# Supplementary material for: Overexpression of AcWRKY31 Increases Sensitivity to Salt and Drought and Improves Tolerance to Mealybugs in Pineapple
Source: Plants (Basel). 2024 Jul 5;13(13):1850. doi: 10.3390/plants13131850 (PMC11243833; doi:10.3390/plants13131850)
Supplement: Supplementary file 1 [file plants-13-01850-s001.zip › Supplementary Figures.pdf]

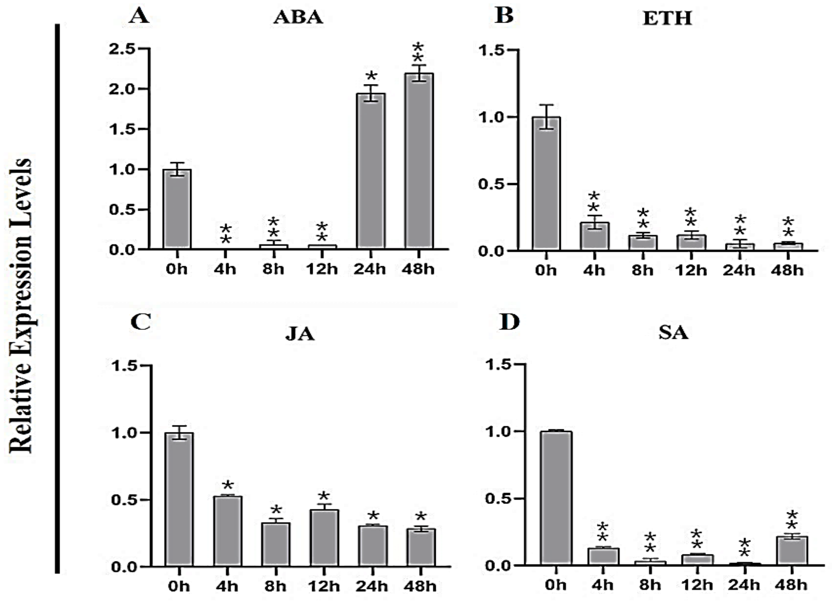

Supplementary Figure S1. Relative expression levels of *AcWRKY31* in different hormone treatments (A) Abscisic Acid (ABA) (B) Ethylene (ETH) (C) Jasmonate Acid (JA) and (D) Salicylic Acid (SA).

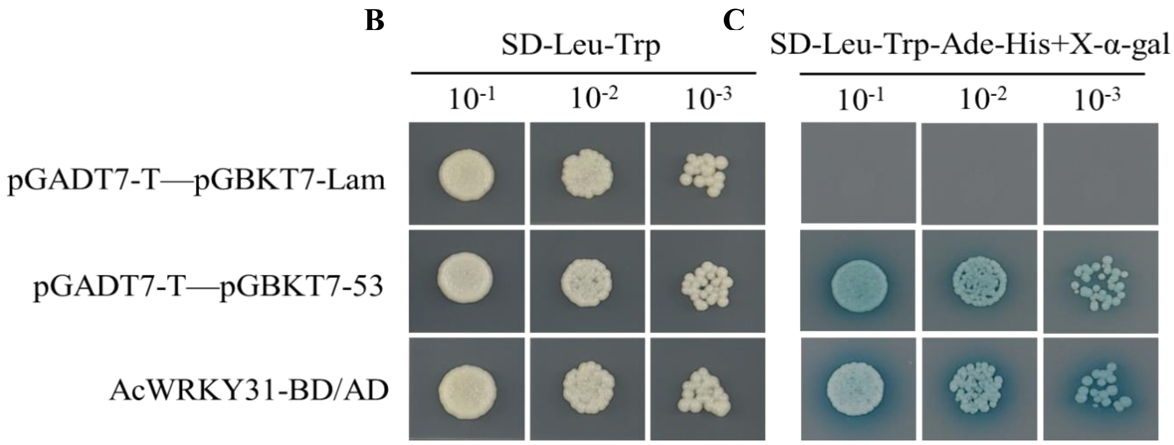

Supplementary Figure S2. Analysis of transcriptional activation activity of *AcWRKY31* (A) The growth of *AcWRKY31*-BD /AD, negative and positive control yeast on SD-Leu-Trp. (B) The growth of *AcWRKY31*-BD/AD and negative-positive co-transformed yeast on SD-Leu-Trp-Ade-His+X-α-gal medium

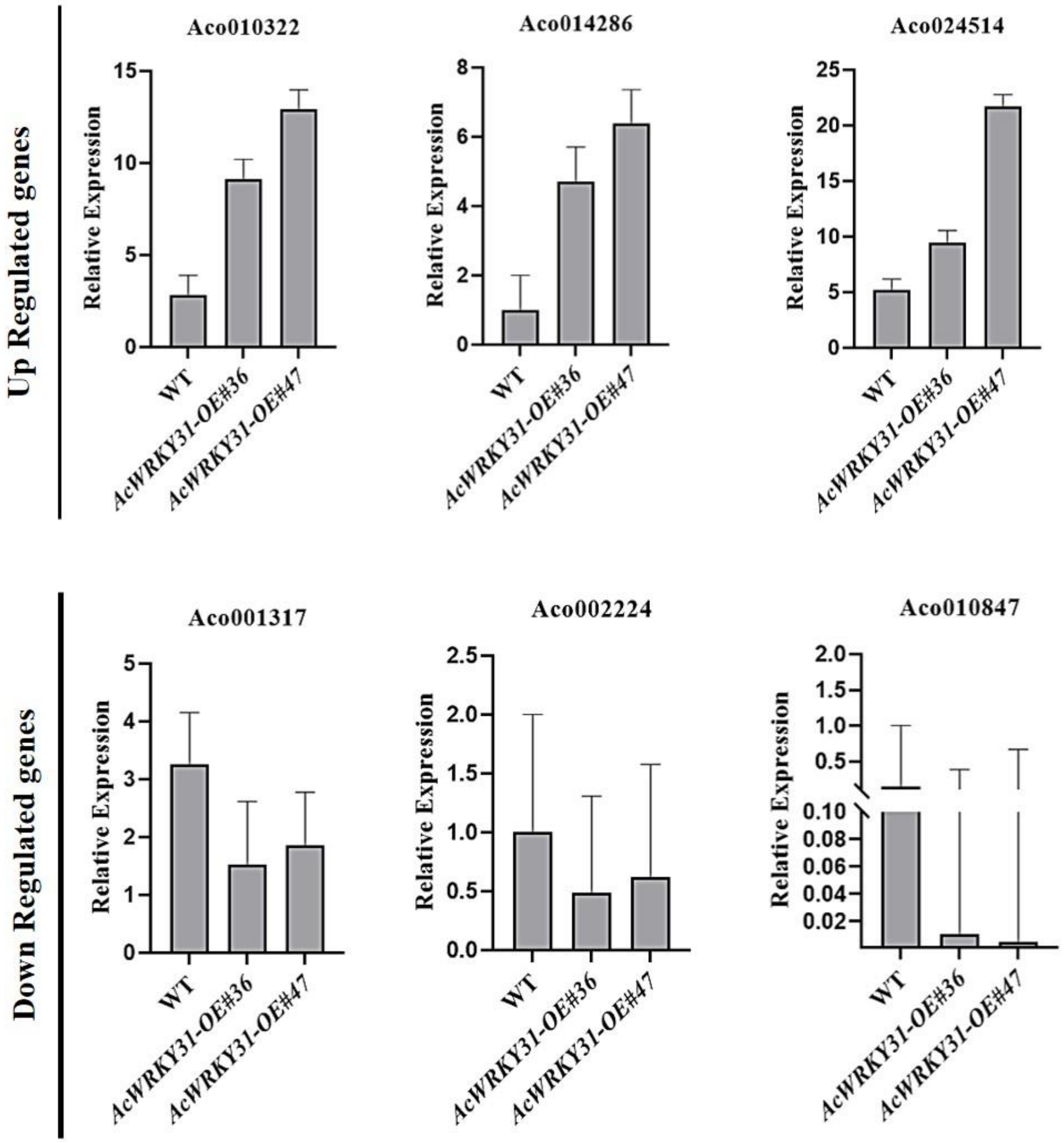

Supplementary Figure S3. Relative expression levels of Up Regulated Genes and Down Regulated Genes from RNA sequence analysis of WT and transgenic pineapples lines, the error bars indicate ± SD ($n = 3$)

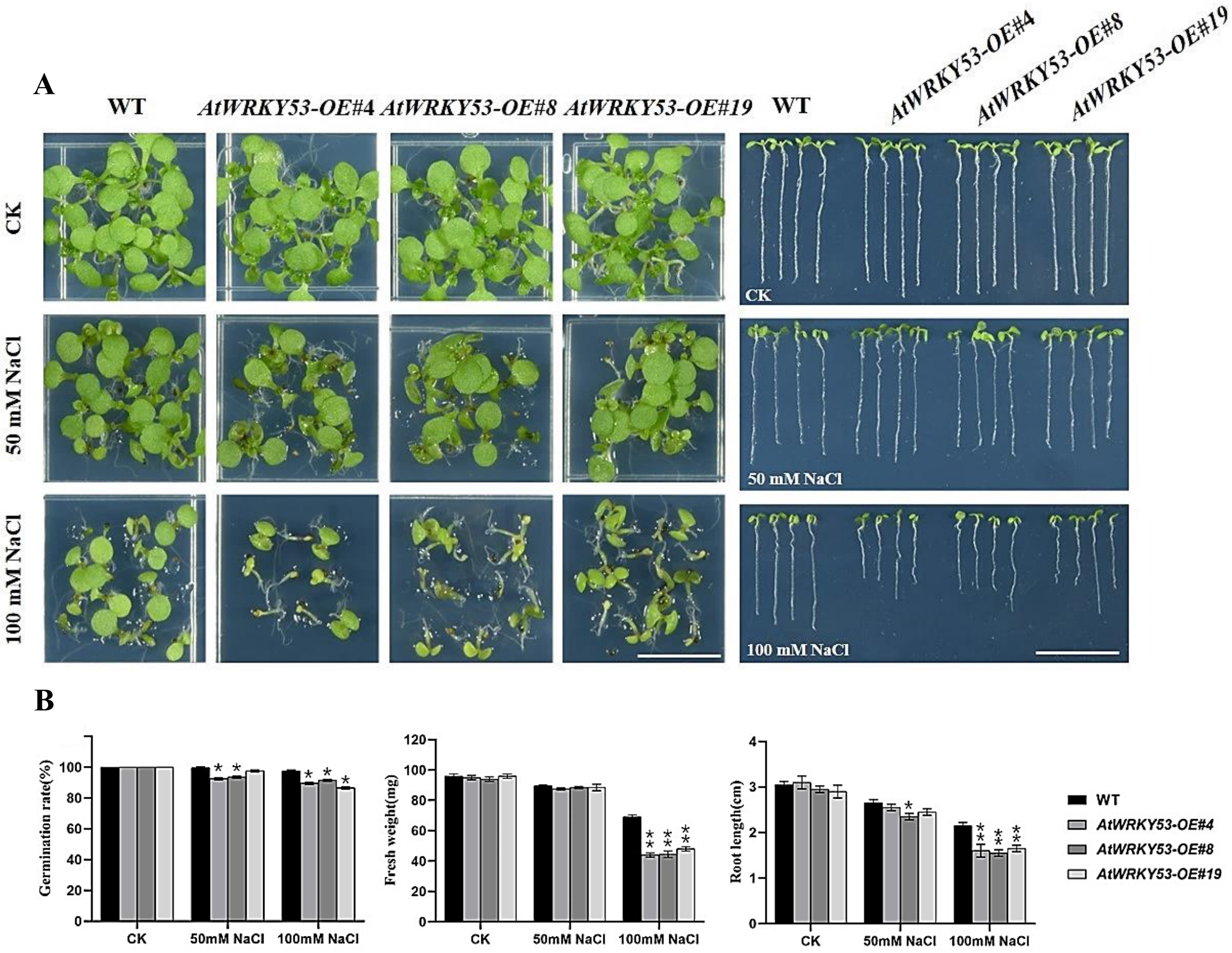

Supplementary Figure S4. Salt treatment on *AcWRKY53-OE Arabidopsis* (A) Observation of germination rate and root length, bar = 1 cm (B) Statistical results of fresh weight and root length of *AcWRKY53-OE* and WT *Arabidopsis* after 7 days of NaCl treatment, the error bars indicate ± SD (n = 3) the asterisks indicate the different significances based on Student's T-test (**p <0.01,*p<0.05)

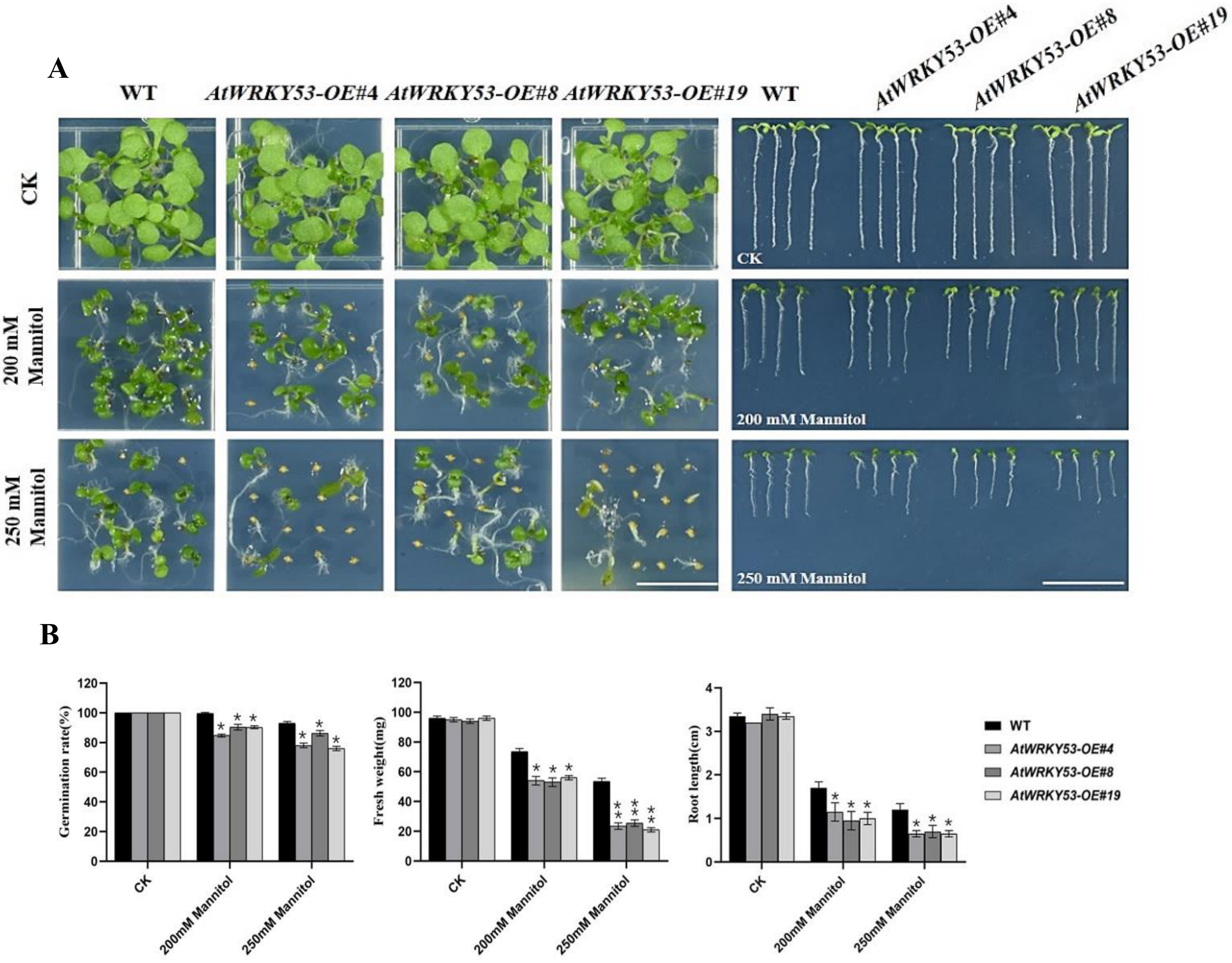

Supplementary Figure S5. Drought treatment on *AcWRKY53-OE Arabidopsis* (A) Observation of germination rate and root length, bar = 1 cm (B) Statistical results of fresh weight and root length of *AcWRKY53-OE* and WT *Arabidopsis* after 7 days of Mannitol treatment, the error bars indicate ± SD (n = 3) the asterisks indicate the different significances based on Student's T-test (**p <0.01,*p<0.05)

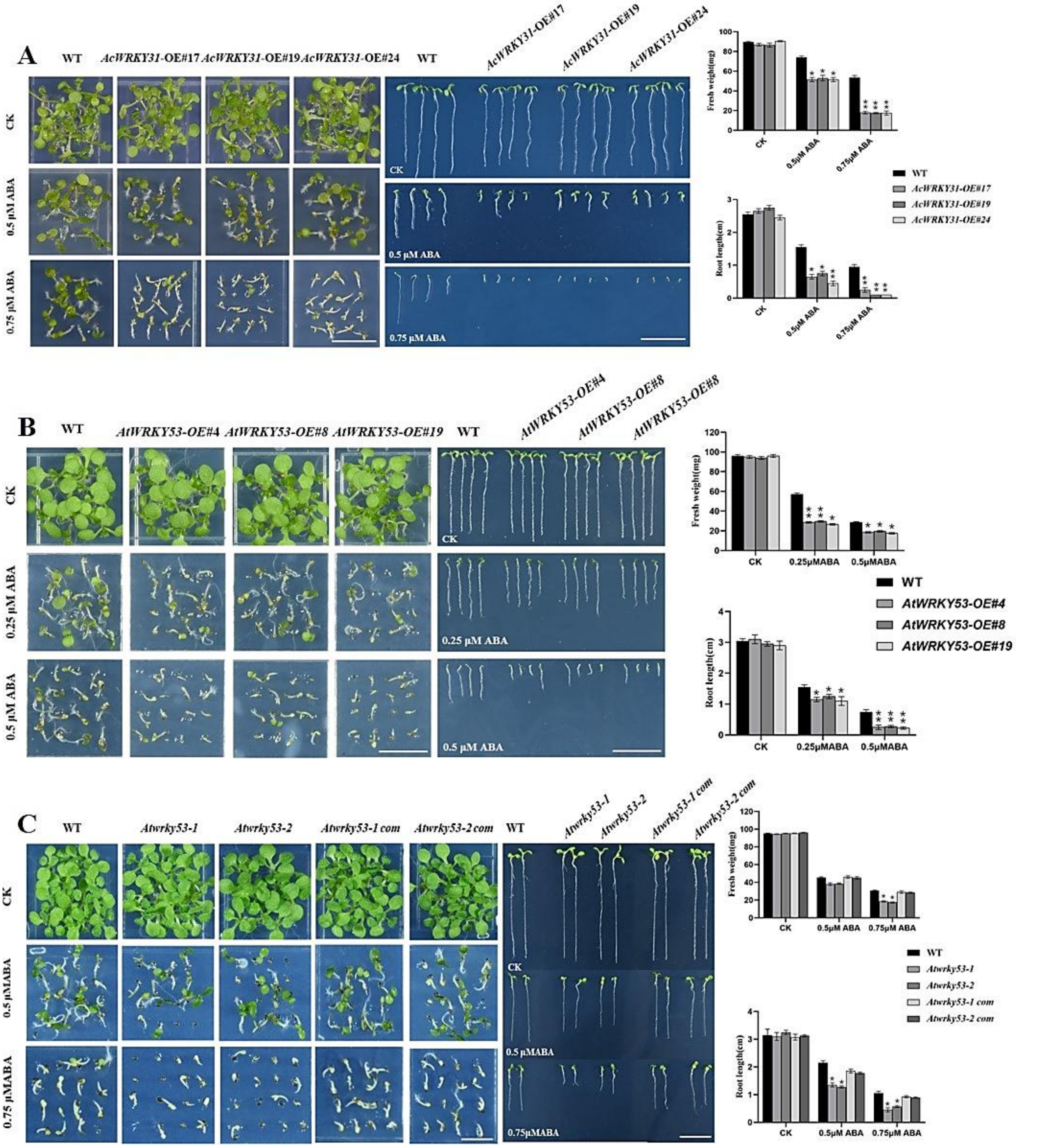

Supplementary Figure S6. ABA treatment on *Arabidopsis* (A) Observation of germination rate, fresh weight, and root length of *AcWRKY31-OE* and WT *Arabidopsis* (bar = 1 cm) (B) Observation of germination rate, fresh weight, and root length of *AtWRKY53-OE* and WT *Arabidopsis* (bar = 1 cm) (C) Observation of germination rate, Fresh weight and root length of *Atwrky53* mutant, complementary and WT *Arabidopsis* (bar = 1 cm); Statistical results of fresh weight and root length after 7 days (Notes: the error bars indicate ± SD (n = 3) the asterisks indicate the different significances based on Student's T-test (**p <0.01,*p<0.05))
